# Transcriptomic Analysis of Changes in Gene Expression During Flowering Induction in Sugarcane Under Controlled Photoperiodic Conditions

**DOI:** 10.3389/fpls.2021.635784

**Published:** 2021-06-15

**Authors:** João Ricardo Vieira Manechini, Paulo Henrique da Silva Santos, Elisson Romanel, Michael dos Santos Brito, Maximiliano Salles Scarpari, Stephen Jackson, Luciana Rossini Pinto, Renato Vicentini

**Affiliations:** ^1^Laboratório de Biologia de Sistemas, Departamento de Genética, Evolução, Microbiologia e Imunologia, Instituto de Biologia, Universidade Estadual de Campinas (UNICAMP), Campinas, Brazil; ^2^Departamento de Genética e Melhoramento de Plantas, Faculdade de Ciências Agrárias e Veterinárias, Universidade Estadual de São Paulo (UNESP), Jaboticabal, Brazil; ^3^Laboratório de Genômica de Plantas e Bioenergia (PGEMBL), Departamento de Biotecnologia, Escola de Engenharia de Lorena (EEL), Universidade de São Paulo (USP), Lorena, Brazil; ^4^Instituto de Ciência e Tecnologia, Universidade Federal de São Paulo (UNIFESP), São José dos Campos, Brazil; ^5^Centro de Cana, Instituto Agronômico de Campinas (IAC), Ribeirão Preto, Brazil; ^6^School of Life Sciences, The University of Warwick, Coventry, United Kingdom

**Keywords:** sugarcane, flowering, photoperiodism, transcriptome, artificial induction

## Abstract

Flowering is of utmost relevance for the agricultural productivity of the sugarcane bioeconomy, but data and knowledge of the genetic mechanisms underlying its photoperiodic induction are still scarce. An understanding of the molecular mechanisms that regulate the transition from vegetative to reproductive growth in sugarcane could provide better control of flowering for breeding. This study aimed to investigate the transcriptome of +1 mature leaves of a sugarcane cultivar subjected to florally inductive and non-inductive photoperiodic treatments to identify gene expression patterns and molecular regulatory modules. We identified 7,083 differentially expressed (DE) genes, of which 5,623 showed significant identity to other plant genes. Functional group analysis showed differential regulation of important metabolic pathways involved in plant development, such as plant hormones (i.e., cytokinin, gibberellin, and abscisic acid), light reactions, and photorespiration. Gene ontology enrichment analysis revealed evidence of upregulated processes and functions related to the response to abiotic stress, photoprotection, photosynthesis, light harvesting, and pigment biosynthesis, whereas important categories related to growth and vegetative development of plants, such as plant organ morphogenesis, shoot system development, macromolecule metabolic process, and lignin biosynthesis, were downregulated. Also, out of 76 sugarcane transcripts considered putative orthologs to flowering genes from other plants (such as *Arabidopsis thaliana*, *Oryza sativa*, and *Sorghum bicolor*), 21 transcripts were DE. Nine DE genes related to flowering and response to photoperiod were analyzed either at mature or spindle leaves at two development stages corresponding to the early stage of induction and inflorescence primordia formation. Finally, we report a set of flowering-induced long non-coding RNAs and describe their level of conservation to other crops, many of which showed expression patterns correlated against those in the functionally grouped gene network.

## Introduction

Flowering is an essential part of the life cycle for angiosperms. It influences the plant’s adaptation to different environments, vegetative development, biomass accumulation, and grain production (Hill and Li, 2016). Flowering is coordinated by a diversity of genes organized in an intricate network of five gene pathways: photoperiod, vernalization, autonomous, gibberellin, and age (Wellmer and Riechmann, 2010; Srikanth and Schmid, 2011; Yamaguchi and Abe, 2012; Song et al., 2015; Hill and Li, 2016). These pathways, coupled with soil quality, water supply, and temperature, can restrain, promote, or disrupt flowering (Jackson, 2009; Hong and Jackson, 2015; Brambilla et al., 2017). Genes and metabolic pathways involved with flowering in grasses and in the model plant *Arabidopsis thaliana* share some similarity.

As an important cash crop, sugarcane (*Saccharum* spp. hybrid) supplies most of the global demand for sugar and ethanol and is impacted by flowering, which is highly undesired in the field as the transition from the vegetative to the reproductive stage ceases plant vertical growth and consumes its energetic reservoir, leading to short and underdeveloped stalks as well as a drastic reduction in extractable sugars available for the industry, hence reducing productivity (Berding and Hurney, 2005). On the other hand, flowering is desirable for plant breeding as crosses require both parent plants to have emitted a well-formed inflorescence at the same time. The difficulty of achieving flowering synchronism hampers breeding as sugarcane cultivars show high variability for flowering time (Glassop et al., 2014; Melloni et al., 2015).

The genus *Saccharum* accounts for a variety of species spread across several countries in five continents and possesses a wide range of photoperiodical behaviors (Moore and Berding, 2013) and multiple critical day lengths (CDL) required for flowering. Sugarcane cultivars, for instance, are considered as intermediate day length plants (IDP), meaning that they can flower around the CDL with many levels of floral induction, requiring a critical photoperiod of approximately 12 h 55 min with daily reductions of 45 s in artificial photoperiod regimes to promote flowering, characterizing a quantitative short day behavior (Moore and Berding, 2013; Glassop et al., 2014; Melloni et al., 2015). Photoperiod facilities have been used successfully in this crop to induce flowering (Melloni et al., 2015; Hale et al., 2017), allowing the control of important external factors that affect flowering, such as temperature, humidity, and day length, thus enabling the simulation of ideal conditions for flowering induction.

Photoperiodic flowering is controlled by a subset of genes involved with photo-perception, circadian rhythm, and molecular long distance signaling (McWatters et al., 2001; Putterill and Varkonyi-Gasic, 2016). At the terminal point of the photoperiodic flowering pathway lies the florigen protein known as FLOWERING LOCUS T (FT), a phosphatidylethanolamine-binding protein (PEBP) expressed in the phloem companion cells following induction by the CONSTANS (CO) protein (Suárez-Lopez et al., 2001; Torti et al., 2012; Wang et al., 2016; Xu et al., 2016). When expressed, the FT protein travels to the shoot apical meristem (SAM), where it interacts with FLOWERING LOCUS-D (FD), a bZIP transcription factor, switching the plant from vegetative to reproductive development by activating the floral meristem identity genes (Andrés and Coupland, 2012). For that to happen, *CO* needs to be induced by the GIGANTEA (GI) protein, which is itself repressed by the gene *CYCLING DOF FACTOR 1* (*CDF1*). Hence, the latter must be repressed by the combined action of genes *FLAVIN-BINDING, KELCH REPEAT, F-BOX 1* (*FKF1*), *GI*, and *PSEUDO-RESPONSE REGULATOR* (*PRR*) *5, 7*, and *9* (Huq et al., 2000; Imaizumi et al., 2005; Mizuno and Nakamichi, 2005; Seaton et al., 2015; Yuan et al., 2019). These *PRR* genes can be activated by expression of a MYB-related transcription factor called LATE ELONGATED HYPOCOTYL (LHY), which, in turn, is activated by photoreceptor transcription factor PHYTOCHROME INTERACTING FACTOR 3 (PHY3) while under red light exposure (Martínez-García et al., 2000; Mizuno and Nakamichi, 2005; Lu et al., 2009). Aside from genes expressing their respective proteins, there is also post-translational regulation caused by interactions with other RNA species, such as microRNAs (miRNA) and long non-coding RNAs (lncRNA) (Yamaguchi and Abe, 2012; Henriques et al., 2017; Zhu et al., 2017). In Arabidopsis, Schmid et al. (2003) show the role of the microRNA miR172 precursor gene family in the photoperiodic induced flowering, mediating the accumulation of *AP2*-related gene mRNA. Over the last decade, lncRNA research has grown rapidly since the discovery of their ability to modulate gene expression levels. However, most of their functions remain unknown due limitations of *in silico* detection (Budak et al., 2020).

Despite the importance of sugarcane flowering for the sugar-energy sector, a deeper understanding of the regulations responsible for the photoperiodic induction process is still scarce (Coelho et al., 2013, 2014; Glassop and Rae, 2019), and the large-scale investigation of differentially expressed (DE) genes of a cultivar under controlled conditions of photoperiodic induction, in fact, still needs to be reported. Studies have been conducted with data mined from the Sugarcane Expressed Sequence Tag (EST) Project (SUCEST) database in order to identify putative flower-specific genes in sugarcane via homology to *Arabidopsis thaliana* flower development genes and proteins (Figueiredo et al., 2001), specifically the multigene family of MADS-box transcription factors and the gene family *APETALA2* (*AP2*), a key gene for flower development that acts as a promoter of early floral meristem identity (Dornelas and Rodriguez, 2001). Most of the studies of gene expression related to sugarcane flowering were carried out using plant material collected in the field after floral induction, a biological material that does not reflect the period before flowering or of flowering induction itself (Papini-Terzi et al., 2005; Medeiros et al., 2016).

The RNA sequencing technique (RNA-Seq) established prominence in high-throughput analysis of transcriptomes for literally every organism, allowing the measurement of transcript expression levels much more accurately when compared with other methods, e.g., Northern blot, ESTs, and microarrays (Wang et al., 2009; Nonis et al., 2014; Das et al., 2020). The RNA-Seq technique can also detect small sequence variations, such as SNPs and regulatory elements, such as non-coding and lncRNAs (Vicentini et al., 2012; Ilott and Ponting, 2013; Cardoso-Silva et al., 2014; Veneziano et al., 2016). In the case of sugarcane cultivars, having a huge, aneupolyploid, and highly complex genome (Garsmeur et al., 2018; Mancini et al., 2018), the use of the RNA-Seq technique is also beneficial for bypassing certain challenges regarding data assembling and analysis.

In the present work, we describe the transcriptomic profiling of a mature leaf during photoperiodic induction of flowering of a sugarcane commercial cultivar. We identify 7,083 DE genes, of which 5,623 showed significant identity to other plant genes. Nine of these genes, related to flowering and response to photoperiod, identified in our RNA-Seq experiment, were analyzed via RT-qPCR either at mature or spindle leaves at two development stages corresponding to the early stage of induction and inflorescence primordia formation. We also investigate the presence and conservation of photoperiodic induced/repressed lncRNAs that may integrate the regulation module for flowering in sugarcane.

## Materials and Methods

### Plant Material and Photoperiodic Treatments

The IAC sugarcane cultivar IACSP96-7569 has a regular flowering behavior under artificial photoperiodic regimes (Melloni et al., 2015). This cultivar was vegetatively propagated by single bud chips, planted into boxes filled with substrate (Plantmax^®^) and placed in a greenhouse for 28 days. Plantlets were transferred to 3.8-L tree pots filled with equal amounts of clay soil, sand, and substrate (Plantmax^®^) and placed randomly in two rail carts of an automated photoperiod facility at the Centro de Cana – IAC at Ribeirão Preto, São Paulo state, Brazil. After 7 months, plants were submitted to two different photoperiod treatments (one for each rail cart), either a constant long day (LD) photoperiod of 13 h and 30 min for non-inductive photoperiodic treatments (NIPT), or a short day (SD) photoperiodic treatment of 12 h and 50 min shortened by 45 s per day as an inductive photoperiodic treatment (IPT) until inflorescence emergence. Once a week, +1 mature leaf (first leaf with visible dewlap), the spindle leaves (furled immature leaves), and the SAM were collected from three plants (biological replicates) in each photoperiodic treatments (six plants total) at ZT10 (Supplementary Figure 1). The +1 mature leaf and the spindle leaves collected from the same plant were immediately frozen in liquid nitrogen and later stored at −80°C until RNA extraction, and the corresponding SAM was fixed in FAA 50% (formalin, acetic acid, and ethyl alcohol) for histological sectioning to confirm the apical meristem developmental stage. The IPT SAM samples, corresponding to the seventh week (45 days after the beginning of the photoperiodic treatment) showed start of transition from vegetative to floral meristem through histological assessment (Supplementary Figure 2). Hence, the +1 mature leaf samples of this time point, in both IPT and NIPT, were selected for RNA-Seq analysis to assess DE transcripts between IPT and NIPT.

### RNA Extraction, Quantification, Sequencing, and Data Set Quality Control

Total RNA extraction was performed by using the PureLink RNA Mini Kit (Thermo Fisher Scientific^®^) according to the manufacturer’s recommendations. RNA Integrity Number (RIN ≥ 8.0) and quantity were evaluated using a 2,100 Bioanalyser (Agilent^®^) spectrophotometer. Libraries were assembled according to mRNA-Seq Sample Preparation v2 kit (Illumina^®^) recommendations. Briefly, mRNA was isolated and annealed to primers for single-stranded cDNA synthesis. The mRNA template was removed, and the second cDNA strand was synthesized, generating the double-stranded cDNA (dscDNA). The dscDNA fragments were isolated and the cDNA fragments ligated to the sequencing adapters. Fragments of 100 bp were amplified through PCR. Finally, the cDNA extracted from six biological replicates (three IPT and three NIPT) collected in the seventh week were taken to the Central Laboratory for High Performance Technologies (LaCTAD – UNICAMP, Campinas, São Paulo state, Brazil) for sequencing in a HiSeq 2,500 (Illumina^®^) sequencer. The FastQC v0.11.7 (Andrews, 2010) and NGS QC Toolkit v2.3.3 (Patel and Jain, 2012) software were used for quality check, adapter/barcode removal, filtering, and trimming procedures. The raw sequence data were deposited in the NCBI SRA database with the accession number SRP302030.

### Reference-Based Mapping and DE Analysis

Transcriptome mapping and gene expression analysis were conducted using BowTie2 v2.3.4.2 (Langmead and Salzberg, 2012) and RSEM v1.3.0 (Li and Dewey, 2011), respectively, with default RSEM parameters for BowTie2, and using a reference sugarcane transcriptome for transcript mapping. Count matrices obtained by RSEM for each library (expected counts, non-normalized) were submitted to DE analysis with a DESeq2 (Love et al., 2014) (*p*-value < 0.05) default script, and the fold change values were internally transformed to log_2_ scale.

### Transcriptome Annotation by Local Alignment and Phylogenetic Analysis

Transcript annotation was performed using BLAST v2.7.1+, retrieving information through similarity analysis with related organisms through a subtractive approach (Supplementary Figure 3). First, we chose four closely related grass protein databases: *Sorghum bicolor* v3.1.1 (McCormick et al., 2018), *Zea mays* v3 (Schnable et al., 2009), *Triticum aestivum* v2.2 (Mayer et al., 2014), and *Oryza sativa* v7.0 (Ouyang et al., 2007) and eudicot model plant *Arabidopsis thaliana* TAIR10 (Lamesch et al., 2012), available at JGI’s Phytozome v12^1^. A BLASTx was performed from the taxonomically closest to the farthest organism from sugarcane with 20 hits per transcript (*e*-value ≤ 1e-05). Hits and no hits were filtered at each BLASTx run. We considered as “hits” the transcripts that produced alignments that passed the filtering parameters. On the other hand, “no hits” are any transcript that either produced alignments that did not pass the filtering parameters or had no alignments produced during BLASTx. Hence, only “no hits” were carried further to the next BLASTx step with the next closest organism, following this order and filter parameters (alignment coverage and alignment identity, respectively): *S. bicolor* (60% and 50%), *Z. mays* (50% and 50%), *T. aestivum* (50% and 50%), *O. sativa* (50% and 50%), and *A. thaliana* (50% and 40%).

Phylogenetic analysis and putative orthology inference were performed by a pipeline (Bottcher et al., 2013) consisting of a series of local and global alignments of target protein sequences taken from KEGG circadian rhythm pathway (McWatters et al., 2001) against our transcriptome and other protein databases (same used previously for *S. bicolor*, *O sativa*, and *A. thaliana*) for phylogenetic relationship inference (Supplementary Table 1). First, target sequences in protein FASTA format were tBLASTn against our transcriptome (identity > 40% and coverage > 50%) capturing the first 15 best hits for each target. Then, these best hits were BLASTp against sorghum, rice, and Arabidopsis protein databases, collecting the best 40 sequences (*e*-value < 1e-05) for a global multiple alignment done with the software MAFFT (Yamada et al., 2016) using default parameters. Finally, these alignments were clustered under maximum likelihood phylogeny analysis conducted by the software phyML (Anisimova and Gascuel, 2006) with a WAG plus gamma substitution model and aLRT test. Results are phylogenetic trees, depicting the target sequences from sorghum, rice, Arabidopsis, and their respective sugarcane transcripts, which show statistically robust evidence for orthology inferring between sugarcane and other plant genes.

### Quantitative Real-Time PCR (RT-qPCR) Assessment of Target Genes

Nine DE transcripts detected in the RNA-Seq experiment and involved in photoperiod response and flowering time were selected for relative gene expression analysis by quantitative real-time PCR (RT-qPCR). These transcripts not only were evaluated at the seventh week (corresponding to RNA-Seq experiment; 45 days of photoperiodic treatment) but also at the 13th week (plants with visible floral primordia formation, after 86 days of treatment; Supplementary Figure 2) in both mature and spindle leaves. Oligonucleotide primer pairs (Table 1) were designed from the respective transcript sequences by using PrimerQuest^®^ Tool^2^ adopting as conditions primer length from 17 to 22 bp; GC content from 35% up to 65%; melting temperature between 59°C and 65°C and amplicon size between 100 and 250 bp. Primer quality was assessed with NetPrimer^3^ and primer pair efficiency by using the software LinRegPCR v7.5 (Ruijter et al., 2009). The cDNA synthesis was performed with the QuantiNova Reverse Transcription kit (QIAGEN Strasse 1, 40724 Hilden, Germany). The RT-qPCR assays were performed using a Bio-Rad IQ5 machine. The reaction was conducted in a final volume of 10 μl containing 5 μL of SYBR Green 2x from GoTaq^®^ qPCR Master Mix Kit, (Promega, United States), 3 μL of cDNA (1:20 dilution), and primer pairs at their respective adjusted concentration. Amplification conditions were 95°C for 3 min, followed by 40 cycles of 10 s at 95°C and 30 s at 60°C, followed by a melting curve from 55°C to 95°C. For the RT-qPCR of the target genes, the three biological replicates sampled in each photoperiodic treatment (IPT and NIPT) were performed in triplicate (i.e., three technical replicates) and technical duplicates adopted for the primer efficiency and optimization analyses. Sugarcane *Ubiquitin1* (*ScUBQ1*) and *Tubulin* (*ScTUB*) were used as normalizers (Table 1). Relative expression data and statistical analysis were performed using the software REST 2009 (Pfaffl et al., 2002) with 2,000 iterations and differences considered significant when *P* < 0.001, *P* < 0.01, and *P* < 0.05. The IPT was considered “treated” and NIPT considered “untreated” for the purpose of expression calculations in the software.

**TABLE 1:** Sugarcane putative photoperiodic response and flowering time genes selected for gene expression analysis by RT-qPCR using mature and spindle leaves collected in the seventh and 13th weeks.

Gene (symbol)	Gene name	Primer sequence (5′- 3′)	Amplicon size (bp)
*ScAGL7*	*AGAMOUS LIKE-7*	F: GACGGTTCAGGCTCAGATT	152
		R: GCTTTAATGAACGCACACCTC	
*ScAGL12*	AGAMOUS	F: GAGATGGGCTATTCCTTCTGAC	149
	LIKE-12	R: CTCCTGAAGGGCTATGGTTTAT	
*ScCDF2*	CYCLING DOF	F: CTGTGATGGTGCCAGGTAAA	147
	FACTOR 2	R: GCACAAGTGGGTATGGAAATG	
*ScCDF3*	CYCLING DOF	F: TCAGGTTTCGACTGGAATGG	151
	FACTOR 3	R: AAGGAGATGAGAAGGCAGAAAG	
*ScEID1*	EID1-like 1	F: TTCTGAGGACACAAAGGAAGAG	166
		R: CAAAGAGAAAGGCAGCTAGGA	
*ScLHY*	LATE ELONGATED	F: GTGTCTCTCCACACAGAGTTAAA	161
	HYPOCOTYL	R: TTGTCCGCATCTACATCACTAC	
*ScPRR1*	PSEUDO-	F: CTCAAGCACATACACCACCA	153
	RESPONSE	R: ATGCCGATGACCACACATT	
	REGULATOR 1		
*ScPRR5*	PSEUDO-	F: ACAGAAGCAGAAACTGACTCG	102
	RESPONSE	R: CCTTCAGTCTTACCAGTCCAAT	
	REGULATOR 5		
*ScPRR7*	PSEUDO-	F: CAGTGGCAGTGGAAGTGAAA	149
	RESPONSE	R: CATTGAGTCCGACACTGAAGTC	
	REGULATOR 7		
ScUBQ1*	UBIQUITIN 1	F: AGCCTCAGACCAGATTCCAA	110
		R: AATCGCTGTCGAACTACTTGC	
ScTUB*	TUBULIN	F: CTCCACATTCATCGGCAACTC	237
		R: TCCTCCTCTTCTTCCTCCTCG	

*Reference genes.

### Gene Ontology Terms Enrichment and Functional Annotation Analysis

Annotation and mapping of Gene Ontology (GO) terms were made with OmicsBox v1.1.164 (BioBam Bioinformatics, 2019) using RefSeq non-redundant release 49 database (Pruitt et al., 2012) to annotate the sugarcane transcriptome. For mapping of GO terms, the GOA database v2019.08 was used. Gene set enrichment was conducted with a Fisher’s test with detailed results for all GO terms for biological processes. Gene sets had a maximum of 4,000 results with a false discovery ratio filter of 0.25. Enrichment networks were rendered with the software Cytoscape (Shannon et al., 2003) with ClueGO (Bindea et al., 2009) using annotated Arabidopsis gene names taken from BLASTx essays. Tree maps were created using REVIGO (Supek et al., 2011). Functional enrichment analysis were conducted with MapMan (Schwacke et al., 2019) using *A. thaliana* mapping “X4 Araport11 R1.0” and pertinent pathways related to flowering, photoperiod, and photosynthesis (downloaded from: https://mapman.gabipd.org/mapmanstore). Sugarcane transcripts annotated against the Arabidopsis TAIR10 database (Lamesch et al., 2012) were used to create an experimental file containing only DE transcripts from sugarcane converted to *A. thaliana* names.

### Analysis of lncRNA

DE lncRNAs (lncRNAs) were identified *in silico* in two steps: First, we classified all “no hit” DE transcripts (*n* = 1,460) identified in our pipeline using CPAT (Wang et al., 2013) to verify the coding and ncRNA probabilities for “no hit” sequences. The program was trained with 400 randomized lncRNAs and 400 coding genes from *Zea mays*, and the threshold to consider ncRNA was >0.3. For the non-coding set, we aligned transcripts against the *S. bicolor* genome using Sim4 (Florea et al., 1998). Second, we classified the lncRNA data set for conservation between other species by using data available in GreeNC (Gallart et al., 2016), which annotates lncRNAs present in genomes available in the Phytozome database, and BLASTn (*e*-value 1e-20) against the following data: *S. bicolor* (*n* = 5305), *Z. mays* (*n* = 18,110), *Setaria italica* (*n* = 3492), *O. sativa* (*n* = 5237), *Brachypodium distachyon* (*n* = 5584), *T. aestivum* (*n* = 38,820), and *A. thaliana* (*n* = 3008).

## Results

### Quality Control of Data, Transcriptome Reference-Based Mapping, Gene Expression Analysis, and Annotation

**FIGURE 1:**
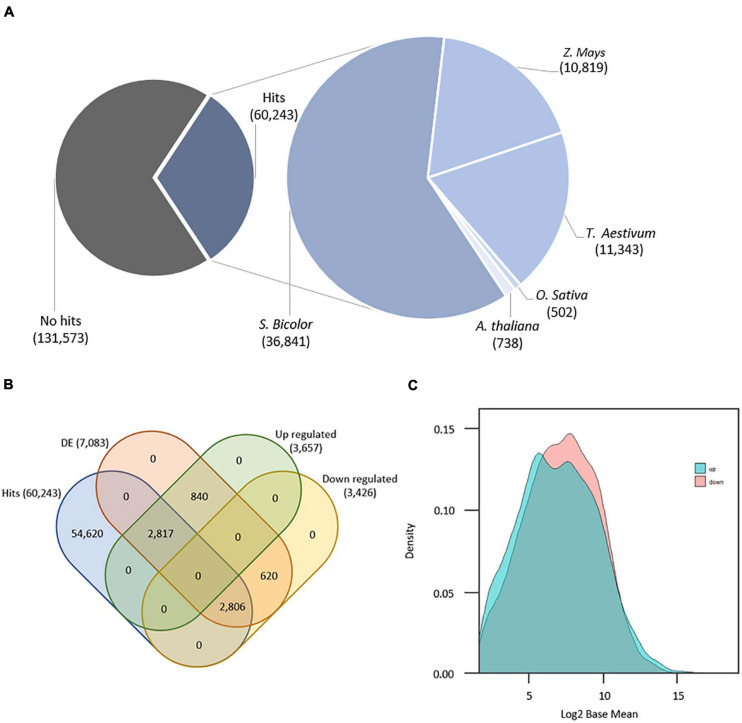
**(A)** Number of annotated transcripts by organism. **(B)** Venn diagram with final transcript amounts after annotation and differential expression analyses. **(C)** Density distribution of differentially expressed genes (*n* = 7,083) by basemean (x) and density (y).

A total of 12 libraries (6 biological samples × 2 technical replicates), encompassing 615,794,012 paired end reads with 100 base pairs (PHRED Score ≥ 30) were produced by RNA-Seq. Quality control of data (i.e., adapter/barcode removal and trimming) resulted in 562,091,180 (91.3% of the total data) high-quality paired-end reads ranging from 70 to 95 base pairs and PHRED score ≥ 38. Transcriptome reference-based mapping resulted in 112,584 mapped transcripts with mean length of 914 base pairs (length < 200 = 26,826 transcripts; > 1.000 = 61,129 transcripts), 63,028 transcripts containing open read frames, and CG content of 48.9% (Supplementary Table 2). Subtractive transcriptome annotation found a total of 60,243 (31.4% of the whole transcriptome) good-quality annotated transcripts (hits): 36,841 annotated from S. *bicolor*, 10,819 from *Z. mays*, 11,343 from *T. aestivum*, 502 from *O. sativa*, and 738 from *A. thaliana* (Figure 1A). Considering a previous study with sugarcane transcriptome annotation (Cardoso-Silva et al., 2014), the present methodology showed an increased number of annotated sugarcane transcripts from *S. bicolor* proteins and reduction for *O. sativa* proteins as the former is closely related to sugarcane. We found 7,083 transcripts with statistically significant DE comparing induced to non-induced treatments. From these, 3,657 are considered upregulated (51.64%), and 3,426 transcripts are considered downregulated (48.36%). There are 2,990 transcripts showing fold change (log_2_) greater or equal to 0.5, and 667 DE transcripts with fold change of less than 0.5 and greater than 0. Downregulated transcripts with fold change less than or equal to −0.5 summed 2,559 with 867 transcripts showing fold change between 0.5 and 0, meaning that there are more DE transcripts either for up- or downregulation at higher values of fold change. We have 5,623 annotated DE genes, being 2,817 upregulated and 2,806 downregulated (Figure 1B). From these, 2,180 upregulated transcripts had fold change greater or equal to 0.5 with 637 having fold change between 0.5 and 0. Also, 1,987 downregulated transcripts showed fold change less than or equal to −0.5, and 819 showed fold change between −0.5 and 0. Finally, transcript density by base mean (i.e., the average value of the normalized counts divided by size factors) of all 7,083 DE transcripts revealed that the downregulated group of transcripts have higher differential expression values than the upregulated group (Figure 1C).

**FIGURE 2:**
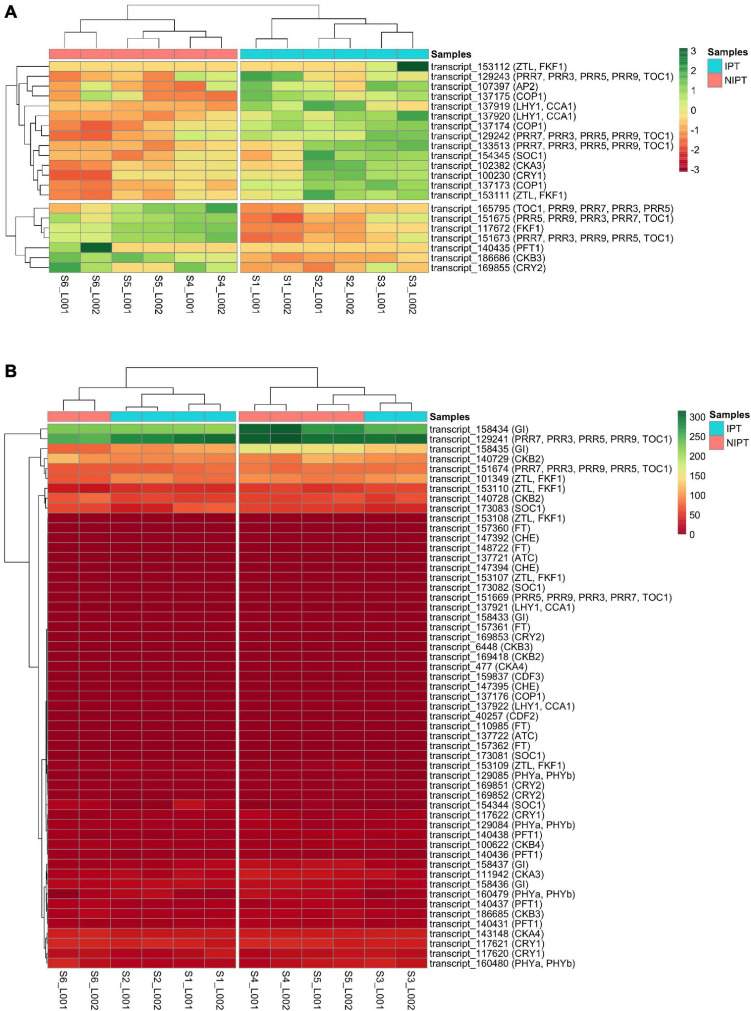
Expression-based clustering of sugarcane orthologs of photoperiodic and flowering genes. Transcripts with **(A)** and without **(B)** DE were color-coded (red to green) by the FPKM values in 12 samples (six for each condition). In panel A, the data were centered and scaled. Euclidean distance was adopted in hierarchical clustering. IPT (Inductive photoperiodic treatment) and NIPT (Non-inductive photoperiodic treatment).

The phylogenetic putative ortholog inference returned 76 transcripts as putative orthologs from *A. thaliana*, sorghum, and rice circadian rhythm and flowering genes as follows (number of putative ortholog transcripts in parenthesis): *ScAP2* (1), *ScATC* (2), *ScCDF2* (1), *ScCDF3* (1), *ScCHE* (3), *ScCKA3* (2), *ScCKA4* (2), *ScCKB2* (3), *ScCKB3* (3), *ScCKB4* (1) *ScCOP1* (4), *ScCRY1* (4), *ScCRY2* (4), *ScFKF1* (1), *ScFT* (5), *ScGI* (5), *ScLHY* (4), *ScPFT1* (5), *ScPHYa* (4), *ScPRR5* (2), *ScPRR7* (6), *ScSOC1* (5), *ScTOC1* (1), and *ScZTL* (7) (Supplementary Table 3). Because we are dealing with sugarcane genes, we renamed our transcripts according to Gray et al. (2009). We detected differential expression for 21 of these transcripts (Figure 2A), of which 14 (*ScAP2*, *ScCKA3*, three *ScCOP1*, *ScCRY1*, two *ScLHY1*, three *ScPRR7*, *ScSOC1*, and two *ScZTL*) were considered upregulated (or induced) and seven (*ScCKB3*, *ScCRY2*, *ScFKF1*, *ScPFT1*, *ScPRR5*, *ScPRR7*, and *ScTOC1*) considered downregulated (or repressed). Interestingly, most of these DE putative ortholog transcripts are related to signaling of light perception (*ScCRY1*, *ScCRY2*, and *ScPFT1*) and to the clock central oscillator (*ScZTL, ScPRR3, ScPRR5, ScPRR7*, and *ScLHY1*) of the circadian rhythm pathway indicating that, in this stage of photoperiodic treatment, the plant is still responding to the photoperiodic stimuli. The remaining 55 detected putative ortholog transcripts showed no DE (Figure 2B). Genes from the canonical flowering pathway, such as *ScFT* and *ScGI*, showed no DE, which was expected as these genes are more active at the start of the signaling stage in the mature leaf before signal translocation and consequent flowering (Kobayashi et al., 1999). These findings indicates that sugarcane, despite already being induced, still regulates the circadian clock pathway in response to photoperiod changes and that the time frame comprehending expression of FT proteins in sufficient quantity, its arrival, and hence accumulation at the SAM to the point of inducing cell differentiation from vegetative to reproductive, in sugarcane, can be short.

### Assessment of Relative Gene Expression by RT-qPCR

**FIGURE 3:**
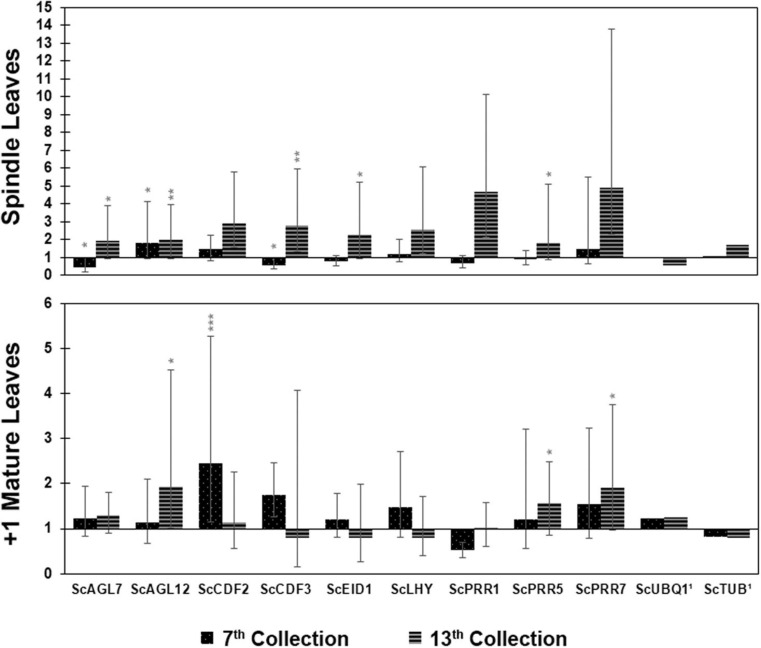
Relative expression (induced/non-induced) of circadian clock genes at two time points following the start of the photoperiodic treatment; seventh week (45 days) and 13th week (85 days), from both spindle leaves and mature leaves. ^1^Reference Genes. *P* < 0.001 (***); *P* < 0.01 (**); and *P* < 0.05 (*).

Gene expression in induced leaves relative to non-induced leaves conducted via RT-qPCR showed that, for spindle leaves collected in the seventh week, the DE genes with statistical significance were *ScCDF3* (0.57), *ScAGL7* (repressed, 0.45), and *ScAGL12* (1.84) (Figure 3). For the 13th week, the DE genes with a statistically significant difference were *ScPRR1* (4.66), *ScLHY* (2.57), *ScAGL12* (1.98), *ScEID1* (2.28), *ScCDF2* (2.89), *ScCDF3* (2.80), *ScPRR5* (1.83), *ScAGL7* (1.94), and *ScPRR7* (4.93), all of them induced. For mature leaves in the seventh week, the DE genes with a statistically significant difference were *ScPRR1* (repressed, 0.51), *ScCDF2* (2.44), and *ScCDF3* (1.75). Finally, for mature leaves of the 13th week, the genes that showed significant differential expression were *ScAGL12* (induced, 1.93), *ScPRR5* (1.56), and *ScPRR7* (induced, 1.90) (Supplementary Table 4).

The dissociation curves in spindle leaves for the seventh week all showed specific PCR amplification of the target gene. Efficiency (E) ranged from 1.37 to 2.03 and *R*^2^ = 0.99. At the 13th week, the *ScPRR1* gene showed a bad dissociation curve, which made its use impracticable. The rest of the genes showed a single peak, indicating specificity. Efficiency ranged from 1.68 to 1.97 and *R*^2^ from 0.77 to 0.99. In the mature leaves, at the seventh week, all primers showed a single peak indicating amplification of a single product. Efficiency varied from 1.66 to 2.04, and *R*^2^ was always maintained at 0.99. The dissociation curves in the mature leaf at the 13th week were also all reasonable, showing a single peak each. Efficiency was 1.68 to 2.2 and *R*^2^ = 0.99 (Supplementary Figures 4–7).

### Gene Ontology Terms Enrichment and Functional Annotation Analysis

**FIGURE 4:**
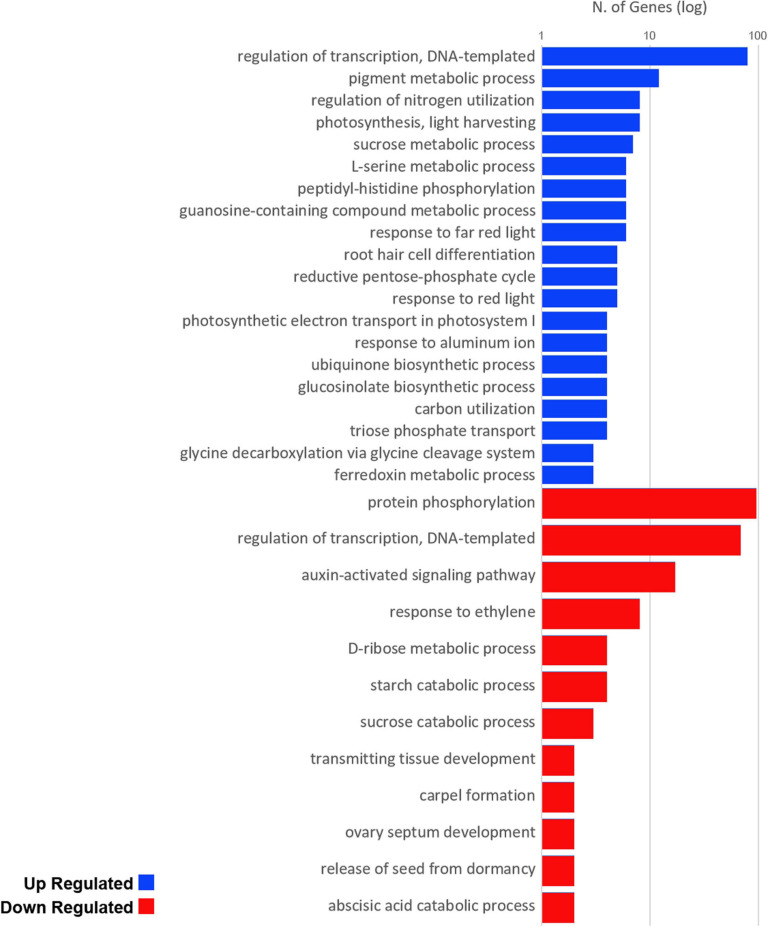
Up (blue) and down (red) regulated overrepresented biological process GO terms present in our data set. Values are number of genes, scaled to log_10_.

Due the reasonably large amount of annotated DE genes detected in our data, we initially resort to large-scale analysis to interpret the overall situation of expressed genes despite the eventual loss of detail. This analysis was conducted individually for the groups of induced (upregulated) and non-induced (downregulated) genes. The present Gene Ontology enrichment returned an extensive set of terms for biological processes with some relation to the literature of flowering in monocots (Coneva et al., 2012; Minow et al., 2018). In the induced gene group, we found upregulation of overrepresented GO terms (Figure 4, with details provided at Supplementary Table 5) with important roles for plant structure development (pigment metabolic process, regulation of nitrogen, and carbon utilization), photorespiration, and energetic metabolism (sucrose, L-serine, and guanosine-containing compound metabolism, triose phosphate transport, ubiquinone biosynthesis, reductive pentose-phosphate cycle, glycine decarboxylation, and ferredoxin metabolism), and photosynthetic activities (photosynthesis/light harvesting, response to red and far-red light, and photosynthetic electron transport in photosystem I) as well as for other responses to external stimuli (glucosinolate biosynthesis and response to aluminum). On the other hand, we found downregulation of overrepresented GO terms related to vegetative growth (auxin-activated signaling pathway, response to ethylene, and transmitting tissue development), energetic metabolism (D-ribose metabolism, starch, and sucrose catabolism), reproductive structure formation (carpel and ovary septum development), and dormancy and seed germination regulation (release of seed from dormancy and abscisic acid catabolism). For a more general representation on how these terms relate, tree maps were generated for easier visualization of these overrepresented GO terms (Supplementary Figure 8).

**FIGURE 5:**
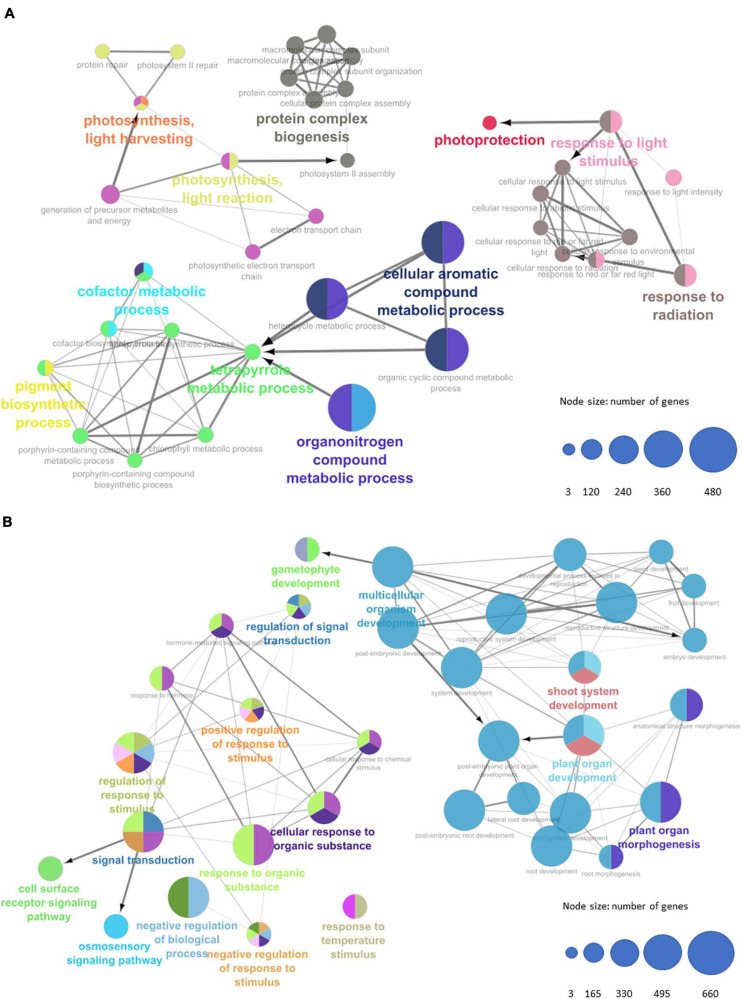
Gene Ontology enriched terms network representing of biological processes for **(A)** upregulated transcripts and **(B)** downregulated transcripts. Node sizes represent number of genes per term (Bonferroni step-down *p*-value < 0.05).

We submitted all GO-enriched terms for functional clustering and annotation with ClueGO to uncover more significant term clusters with a higher degree of term specificity. GO networks revealed thousands of interconnected elements; hence, a *p*-value cutoff filter (<0.05) had to be implemented to capture more significant term clusters (Figure 5). Photosynthetic activity terms are still present at the induced group of genes after cutoff, showing photosynthesis, light harvesting, response to light stimulus, and response to radiation. This cluster induces the protein complex biogenesis through modulation of the photosystem II assembly. In turn, response to light stimulus, light intensity, and radiation seem to be inducing genes related to photoprotection. A third cluster of significantly enriched GO terms for the group of induced genes shows activity of a substantial number of genes (more than 480 genes) related to chemical compound and pigment metabolisms, such as tetrapyrrole and chlorophyl, respectively. As for the non-induced group of genes, two major clusters are portrayed: the first one (left side of Figure 5B) shows GO terms related to signal transduction, response to stimuli, response to chemical stimuli, and response to hormones, which could demonstrate some relation between repression of these response mechanisms during the change from the vegetative to the reproductive stage. Hence, as shown, this cluster may be responsible to modulate cell surface receptor signaling and osmosensory signaling pathways. The second major cluster (right side of Figure 5B) depicts terms related to shoot system development, plant organ morphogenesis, and plant organ development. This cluster might also be involved in the modulation of the post-embryonic plant organ development and as well in gametophyte development, which is expected to be found in +1 mature leaves after floral induction. Furthermore, this can also be an indicator that the time frame between the induction of the FT protein and its critical accumulation in the SAM is short in sugarcane.

**FIGURE 6:**
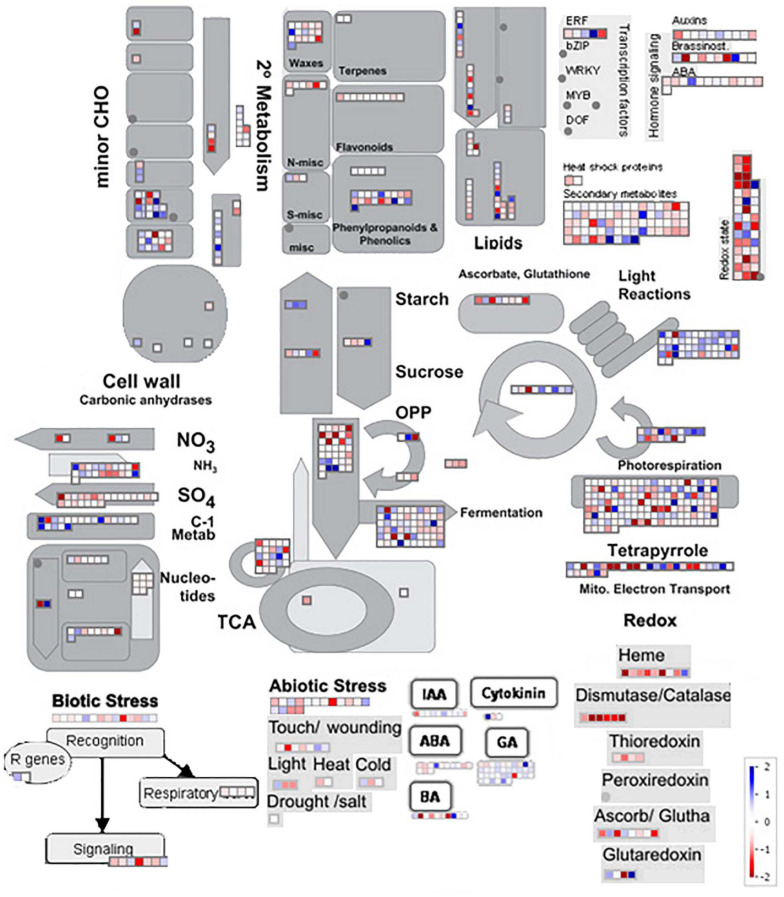
MapMan functional annotation showing parts of the metabolism overview, hormones, biotic stress, abiotic stress, and redox reaction pathways. Color scales from –2 (intense red) to +2 (intense blue), in log2 fold change.

In general, the functional annotation made with MapMan shows expression changes in known gene pathways in plants (Figure 6). The effects of the flowering inductive photoperiod on sugarcane extends throughout several metabolic pathways and regulatory modules of the plant. There are significant gene expression alterations for some pathways, such as minor carbohydrate, starch/sucrose, lipids, and cell wall metabolisms, and hormones. Light reactions and photorespiration pathway alterations are predominant, and 2° metabolism, redox reactions, fermentation, tetrapyrrole metabolism and hormone signaling showed a considerable amount of DE genes. Interestingly, there are some small expression changes for biotic and abiotic stresses: signaling for biotic stress is mostly downregulated, and light and cold biotic stress responses show mixed results though small (log2 fold change < 1 and >−1).

### Sugarcane lncRNAs Responsive in Flowering Induction

**FIGURE 7:**
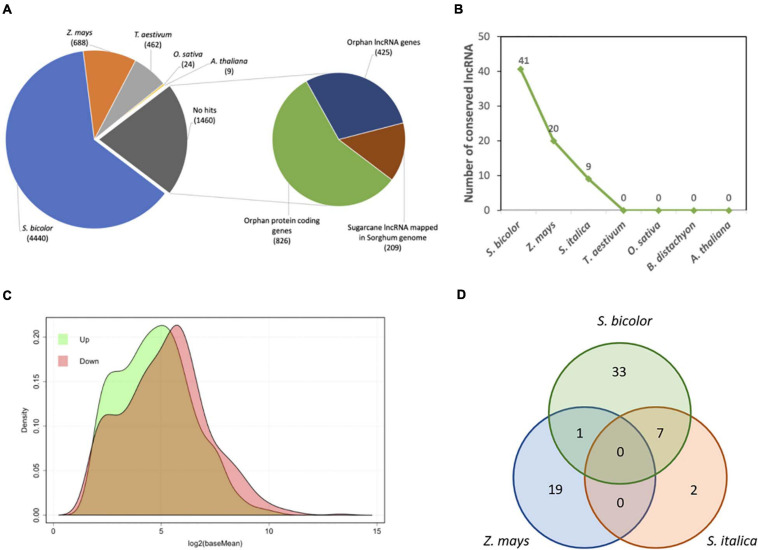
The conservation and expression profile of sugarcane lncRNAs responsive in flowering inducted by photoperiod. **(A**) DE sugarcane transcripts (7,083) classified by annotation pipeline: the 1,460 no-hit transcripts were classified into coding (826), conserved non-coding (209), and orphan non-coding (425) transcripts. **(B)** Number of conserved sugarcane lncRNAs (634) with *S. bicolor*, *Z. mays*, *S. italica*, *T. aestivum*, *O. sativa*, *B. distachyon*, and *A. thaliana*. **(C)** Density distribution of up- (840) and downregulated (620) sugarcane no-hit transcripts. **(D)** Venn diagram with the number of sugarcane lncRNAs showing conservation to related grasses, including shared conservation between species.

We have also made an *in silico* analysis to identifying sugarcane flowering induction responsive lncRNAs. Such RNAs are characteristic for lacking or having small open reading frames and being longer than 200 nucleotides. A long non-coding annotation of sugarcane floral induction transcriptome was performed over all 1,460 DE “no hits” transcripts. The BLASTn, Sim4, and CPAT were used in this pipeline (Figure 7A). Sim4 was used to find putative non-annotated genes/lncRNA (ncRNA), CPAT to verify the coding and ncRNA probabilities, and BLASTn to identify conserved sequences. After applying similarity filters, we found 634 sugarcane lncRNAs responsive to flowering induction (Figure 7A). From those, 62 (Supplementary Table 6) are shared with other grass species (Figure 7B). As some lncRNAs may be involved with flowering induction, these are relevant data for further studies (Figure 7C). The lncRNA annotation procedures reported a low level of sequence conservation against known sorghum or other grass transcripts (Figure 7B) but a high level of conservation in the sorghum genome (Figure 7A) was detected, showing that 209 transcripts could be unknown sorghum lncRNAs. Interestingly, there are seven conserved lncRNA sugarcane transcripts in common with *S. bicolor* and *S. italica* and only one with sorghum and *Z. mays* (Figure 7D). There are no conserved sugarcane transcripts in common with *Z. mays* and *S. italica*.

## Discussion

The results presented here start to shed some light on the changes in gene expression when sugarcane plants switch from vegetative to reproductive development following photoperiodic induction. This has not previously been done for sugarcane. We report more than 112,000 mapped sugarcane transcripts, of which 7,083 show significant DE in response to an IPT. Transcripts were annotated following a stringent protocol and robust statistical methods, aiming to find as much high-quality information as possible, which yielded more than 60,000 fully annotated transcripts. Also, we captured 76 transcripts as being putative real orthologs of flowering genes from different plant species in sugarcane with 21 being responsive to photoperiodic induction as well as 565 lncRNAs also responsive to the florally inductive photoperiod, which have many applications in further molecular studies of the subject.

Plants such as *Arabidopsis thaliana* (thale cress), *Glycine max* (soybean), *Oryza sativa* (rice), *Zea mays* (maize), *Sorghum bicolor* (sorghum), *Triticum aestivum* (wheat), and *Hordeum vulgare* (barley) have been intensively studied in the last decade in regard to their control of flowering (Matsubara et al., 2014; Song et al., 2015; Wolabu et al., 2016; Brambilla et al., 2017) and are still used as models for investigating flowering pathways in related plant species. However, novel molecular functions and interactions can still be discovered in less well-studied organisms as in the case of sugarcane. Despite advances in flowering time regulation in grasses, such as *S. bicolor* (Lin et al., 2016), *Z. mays* (Coneva et al., 2012; Minow et al., 2018), *S. italica* (Doust et al., 2017), and *Brachypodium distachyon* (Higgins et al., 2010), the sugarcane photoperiod pathway still waits for gene identification and functional characterization.

Most florally induced genes are expressed in a tissue-specific manner. Once external stimuli are perceived by the right sensors, the pathway is activated to promote the transition from vegetative to reproductive development in plants (Kiełbowicz-Matuk and Czarnecka, 2014). Our real-time qPCR assays show that, in mature leaves collected in the seventh week (floral induction stage), the *ScPRR1* gene was repressed. In *Arabidopsis*, the *AtPRR* gene family consists of five members (*AtPRR9*, *AtPRR7*, *AtPRR5*, *AtPRR3*, and *AtPRR1*/*TOC1*) (Yamamoto et al., 2003) that are involved in the function of the circadian clock (Mizuno and Nakamichi, 2005). *AtPRR* transcripts start to accumulate after dawn in a specific order (*AtPRR9* – *AtPRR7* – *AtPRR5* – *AtPRR3* – *AtPRR1*) with an interval of 2 to 3 h each, forming a loop expression profile (Makino et al., 2001). Glassop and Rae (2019) found that, in the 24-h cycle, the *ScPRR1* gene in LD was highly expressed in spindle leaves and +1 mature leaf, reaching its maximum expression peak with about 10 h into the light regime, followed by a decrease after 2 h in the dark period. In the present study, with a similar time of collection (16 h 00, ZT10), *ScPRR1* appeared to be repressed in both spindle and mature leaves in the situation of shortening day length. In mature leaves at the 13th week, the *ScPRR* family genes that were significantly expressed were *ScPRR5* and *ScPRR7*, both induced and more expressed in the SD than the LD light regime. It should be noted that in the seventh week, the *ScPRR* feedback loop is at its end because it has a specific order of expression, and *ScPRR1* is the last to show a peak of expression. On the other hand, in the 13th week (floral primordium), the “loop” is at the halfway point because, in this sampling point, *ScPRR7* and *ScPRR5* are being expressed. In spindle leaves, at the 13th week, the *ScPRR7* and *ScPRR5* genes are significantly induced and follow the same pattern of expression as in mature leaves of the 13th week.

The *ScLHY* gene was significantly induced in spindle leaves of the 13th week as in the work by Glassop and Rae (2019). However, according to the authors, the peak of *ScLHY* expression in a 24-h cycle occurs mainly in the dark period, differing from our time of collection, which explains why its expression is at the beginning of the ascent in the present work. In *Arabidopsis*, AGAMOUS-LIKE 12 (*AtXAL1*) is a member of the MADS-box family, which is one of the key components in the floral induction and flowering development network. Tapia-López et al. (2008) suggest that *AtXAL1* is an upstream regulator for *AtSOC*, *AtFT*, and *AtLFY*. In sugarcane, *ScAGL12* was significantly induced in mature leaves collected in the 13th week and in spindle leaves of the seventh and 13th weeks. Tapia-López et al. (2008) also note via *in situ* hybridization of the *AtXAL1* mRNA that the gene is specifically expressed in *Arabidopsis* vascular tissue. There, in the phloem’s companion cells, the mRNA of the *AtFT* gene is expressed and translated, so the *AtFT* protein is translocated to the apical meristem, triggering flowering (Corbesier et al., 2007). Nonetheless, if *AtXAL1* acts upstream of the *AtFT* gene, this observation could only be valid for mature leaves in the 13th week because, in the spindle leaves, the vascular system is not yet fully developed. There is a redundancy between the *AtDof* (DNA-binding with one finger) transcription factors (*AtCDF1*, *AtCDF2*, *AtCDF3*, and *AtCDF5*) as negative regulators of *AtCO* at flowering time in *Arabidopsis* (Imaizumi et al., 2005; Fornara et al., 2009). The *ScCDF3* gene was significantly repressed in spindle leaves of the seventh week and mature leaves in the 13th week, inferring that the former tissue, when induced by photoperiod, is potentially inducing CONSTANS (hence FT) to promote flowering in the nearby SAM. Conversely, in seventh week mature leaves and in spindle leaves of the 13th week, flowering induction is expected to be halted. As for *ScCDF2*, there is induction in both tissues and times; however, there is a 2.5-fold increase in expression in seventh week mature leaves, inferring that *ScCDF2* is repressing flowering through repression of CONSTANS, as well as a threefold increase in expression of the gene in spindle leaves of the 13th week, in which flowering is not expected. This behavior of the *Dof* transcription factors seem to be somehow conserved between sugarcane and Arabidopsis despite the great evolutionary distance. However, the rice *Dof* family, comprising more than 30 elements (Li et al., 2009) act as flowering inductors. The *AtEID1* gene is related to a negative regulation through protein degradation (ubiquitin-dependent proteolysis) of *AtPhyA* dependent pathways (Büche et al., 2000) and appears significantly induced in spindle leaves at the 13th week (when light exposure is the lowest) although it is repressed in the seventh week. In Arabidopsis, the absence of *AtEID1* allows *AtPhyA* stability, responding as a far-red light receptor under continuous irradiation (Dieterle et al., 2001). For sugarcane, this relation between *ScEID1* and *ScPhyA* seems to be maintained.

In a recent study from our group (Santos et al., 2021), we tested reference genes against sugarcane DE orthologs of *AtPIL5*, a gene involved with seed germination, leaf senescence, gibberellin pathway, and light perception, and *AtLHP1* (aka *TFL2, TERMINAL FLOWER 2*, Feng and Lu, 2017), which is an epigenetic regulator of *FT* and *FLC* in Arabidopsis. It was found that, for mature leaves at the photoperiodic flowering induction stage (equivalent to this study’s seventh week), *ScPIL5* is induced, and *ScLHP1* is repressed. Interestingly, the induction of *ScPIL5* may infer a different function of the gene between Arabidopsis and sugarcane because, in the former, *AtPIL5* (*AtPIF1*) represses chlorophyl synthesis in dark periods while suppressing hypocotyl elongation during light time, which is not observed in our Gene Ontology analysis (Figure 5A). Also, the repression of *ScLHP1* is related to diverse gene functions between Arabidopsis and sugarcane (Coelho et al., 2014) as, in the former, this gene is responsible for the maintenance of floral meristem identity during flowering, and for the latter, it may also have yet unknown functions for photoperiodic response of flowering in grasses.

To get a general overview of the effects of theinductive photoperiod in sugarcane, we performed large-scale Gene Ontology enrichment terms analysis for DE annotated transcripts and assessed the overrepresented terms for biological processes (Figure 4). As expected, we found several processes and functions linked, directly or indirectly, to plant structure development, photorespiration, energetic metabolism, and photosynthetic activities. Although it is not possible to infer the exact moment of vegetative/reproductive transition, positive changes in the expression of genes involved in biological processes related to photoperiod and light responses indicates that these pathways are being induced. Upregulation of genes involved in pigment metabolism and photosystem I electron transport suggests that the plant may be working under some form of light-deprivation stress. Downregulation of genes involved in hormone pathways, such as the auxin, ethylene, and abscisic acid, may be a result of the change from vegetative to reproductive development and production of the associated reproductive structures. Interestingly, carpel formation, ovary septum development, and seed release from dormancy were also found in downregulated transcripts.

In the attempt to look closer at the large-scale GO term analysis, we conducted a network clustering of the enriched terms and selected the most significant clusters related to circadian rhythm and flowering. By doing so, it is possible to see hierarchies of GO terms at a higher level of detail (Figure 5). Response to light stimuli (light intensity and quality) upregulated together with photoprotection genes, suggesting once more that the plant may be under a kind of light-deprivation stress. Upregulation of light harvesting and light reaction processes can also be involved in the response of the plant to light deprivation, promoting repair of the photosystem II in the attempt to harvest more light in a scenario of decreasing daytime. These processes are also directly involved in the network for protein complex biogenesis and generation of energy, which suggests that the plant might be investing its energy reservoir in the increase of pigment biosynthesis through the tetrapyrrole metabolic pathway, which accounts for chlorophyll biosynthetic processes (Figure 5A). Henceforth, this data raises a question regarding the existence of crosstalk between the photoperiodic induction of flowering and responses to light-deprivation stress. The need for consistency of the flowering signal throughout the flowering stage in sugarcane is of utmost importance given that the interruption of light stimuli causes sugarcane to abort flowering, leading to floral reversion and malformed inflorescence (Glassop et al., 2014). On the other hand, a wide range of significant GO term clusters of downregulated transcripts is also observed (Figure 5B). Curiously, response to temperature stimulus is downregulated under IPT, whereas the temperature pathway is often combined with the photoperiod for flowering induction under the *FLC/SOC1/FUL* (*FLOWERING LOCUS C, SUPPRESSOR OF CONSTANS1*, and *FRUITFULL*) gene regulation module for eudicots (Bouché et al., 2016), suggesting that maybe there is a thinner interdependency between photoperiodic and temperature pathways for flowering in sugarcane. Downregulation of responses to chemical stimuli and hormones also constitutes a wide GO cluster, suggesting that, although the plant is repressing hormone pathways (i.e., auxin, ethylene, and ABA), there is a desensitization of cellular responses to such compounds, allied to downregulation of signal transduction in order to ensure the change from the vegetative development stage to the reproductive stage. Last, plant organ morphogenesis and gametophyte development are interestingly repressed under the inductive photoperiodic treatment. This may be related to halting vegetative shoot development following induction, which would precede floral organ initiation.

Functional annotation and metabolic pathway enrichment data supports the hypothesis that light-deprivation stress occurs during the photoperiodic induction of flowering sugarcane, together with desensitization of hormonal responses (Figure 6) of auxin, ABA, and 6-BenzylAdenine (BA). Moreover, Cytokinin and GA metabolism appear to be slightly upregulated. Abiotic stresses such as wounding, light, and cold responses, show small changes, but most of them seem to be repressed. As detected in the GO analysis, tetrapyrrole metabolism shows large changes, either induction or repression of some genes, which may infer accentuated modulation in pigment and aromatic compound metabolism. Photorespiration and light reactions also show big changes in gene expression, mostly induced, inferring that the plant is undergoing changes in how light (intensity and quality) is perceived, together with an increase in energy production.

Although our group have previously identified 1,446 sugarcane lncRNA expressed in leaves (Cardoso-Silva et al., 2014), this study reveals a set of 634 sugarcane lncRNAs DE under the photoperiodic condition. Furthermore, we show that 62 (approx. 10%) of these sugarcane transcripts share some level of conservation with gene *loci* from other grasses. Unfortunately, to date, most of them were not characterized, and a thorough study of this subset might prove invaluable in unveiling novel regulatory elements in grasses. Recently, a collection and rich resource of 6,510 lncRNAs were identified in Arabidopsis (Zhao et al., 2018). So far, few were functionally involved in flowering time regulation, such as *COLDAIR* and *COOLAIR* related to the vernalization-mediated epigenetic repression of a MADS-box *AtFLC* (Chekanova, 2015; Budak et al., 2020). Besides this, the overexpression of seven intronic lncRNA from Arabidopsis MADS-box genes activates the expression of their host genes (Liu et al., 2020). On the other hand, however, only FLC homologs antisense transcripts present in various grass species, called in *Brachypodium* spp. as *BdCOOLAIR1* and *BdCOOLAIR2*, were functionally characterized regulating *in cis* the rate of a MADS-box *BdODDSOC2* expression (Jiao et al., 2019). These data suggest further lncRNA physical localization and their co-expression pattern together with its host genes in control flowering time.

Future activities to better understand flowering induction of sugarcane under photoperiodic treatments might focus on the large-scale investigation of different time points of induction aiming to verify key genes activities, compared with already published gene regulatory networks from other plants, and how treatments modulate these networks. Data from circadian rhythm experiments in grasses are not abundant and would greatly contribute to the subject. Furthermore, integration of post-translational regulatory elements on expression data for gene regulatory networks, such as lncRNAs, might provide a deeper understanding of flowering in sugarcane.

## Data Availability Statement

The datasets presented in this study can be found in online repositories. The names of the repository/repositories and accession number(s) can be found below: NCBI, BioProject ID: PRJNA691914 and the following link: http://www.ncbi.nlm.nih.gov/bioproject/691914.

## Author Contributions

LP and RV: conceptualization and project administration. JM, PS, and RV: data curation and formal analysis. LP, SJ, and RV: funding acquisition and supervision. JM, PS, ER, MB, MS, SJ, LP, and RV: investigation. JM, PS, MS, LP, SJ, and RV: methodology. LP, MS, and RV: resources. JM, LP, and RV: visualization and writing – original draft preparation. JM, PS, ER, LP, SJ, and RV: writing – review and editing. All authors contributed to the article and approved the submitted version.

## Conflict of Interest

The authors declare that the research was conducted in the absence of any commercial or financial relationships that could be construed as a potential conflict of interest.

## Footnotes

1 https://phytozome.jgi.doe.gov/pz/portal.html

2 www.idtdna.com/SciTools

3 www.premierbiosoft.com/netprimer

## Supplementary Material

Supplementary Figure 1 Schema of the sugarcane leaf structure, emphasizing the spindle leaves (top red circle), and the first leaf from the top to the bottom of the stalk with clearly visible dewlap (bottom red circle) also known as +1 mature leaf (adapted from Van Dillewijn, C. Botany of sugarcane. Waltham: Chronica Botanica, 1952. 371). **(A)** sagittal section of the upper culm: peeled, **(B)** spindle leaves dissected from the central part of the upper culm, **(C)** shoot apical meristem dissected from the bottom part of the upper culm, and **(D)** +1 mature leaf with red arrow showing the dewlap.

Supplementary Figure 2 Morphology of the shoot apical meristem of sugarcane cultivar IACSP96-7569: **(A)** seventh week, 45 days in inductive conditions; **(B)** seventh week, 45 days in non-inductive conditions; **(C)** 13th week, 85 days in inductive conditions; and **(D)** 13th week, 85 days in non-inductive conditions (Santos et al., 2021).

Supplementary Figure 3 Subtractive approach conducted to annotate sugarcane transcripts. All 191,816 transcripts were considered for annotation using successive BLASTx steps with sorghum, corn, wheat, rice, and Arabidopsis protein databases. Alignments produced in each step were filtered for coverage and identity between query and subject sequences, taking “hit” transcripts out of the initial bulk and carrying further “no hits” for the next BLASTx and repeating this procedure until the last organism, Arabidopsis.

Supplementary Figure 4 Dissociation curves for primer pairs from essays on spindle leaves of the seventh week. E, primer efficiency; *R*^2^, Correlation coefficient.

Supplementary Figure 5 Dissociation curves for primer pairs from essays on spindle leaves of the 13th week. E, primer efficiency; *R*^2^, correlation coefficient.

Supplementary Figure 6 Dissociation curves for primer pairs from essays on mature leaves of the seventh week. E, primer efficiency; *R*^2^, correlation coefficient.

Supplementary Figure 7 Dissociation curves for primer pairs from essays on mature leaves of the 13th week. E, primer efficiency; *R*^2^, correlation coefficient.

Supplementary Figure 8 Tree maps representing enriched categories of biological processes for **(A)** upregulated transcripts and **(B)** downregulated transcripts.

Supplementary Table 1 Target sequences. Gene names (and *Arabidopsis thaliana* TAIR9 entry) used for orthology inference.

Supplementary Table 2 Main results and metrics of the reference-based mapping of RNA-Seq transcripts.

Supplementary Table 3 Complete list with results from the phylogenetic analysis. Sugarcane transcripts were clustered closely to respective target *A. thaliana* sequences with correspondence to *S. bicolor* and *O. sativa* homologous genes.

Supplementary Table 4 Gene relative expression conduced with RT-qPCR essays for two time points (seventh week, 45 days of treatment and 13th week, 85 days of treatment) and two tissues (spindle leaves and mature leaves).

Supplementary Table 5 List of overrepresented enriched GO terms (FDR < 0.05) from sugarcane photoperiodic flowering induction transcriptome.

Supplementary Table 6 List of conserved sugarcane lncRNAs responsive to flowering induction and its orthologs in *S. bicolor*, *Z. mays*, and *S. italica* (sorted by highest to lowest log_2_ fold change).
