# Supplementary figures and images for: Transcriptomic Analysis of Changes in Gene Expression During Flowering Induction in Sugarcane Under Controlled Photoperiodic Conditions
Source: Front Plant Sci. 2021 Jun 15;12:635784. doi: 10.3389/fpls.2021.635784 (PMC8239368; doi:10.3389/fpls.2021.635784)

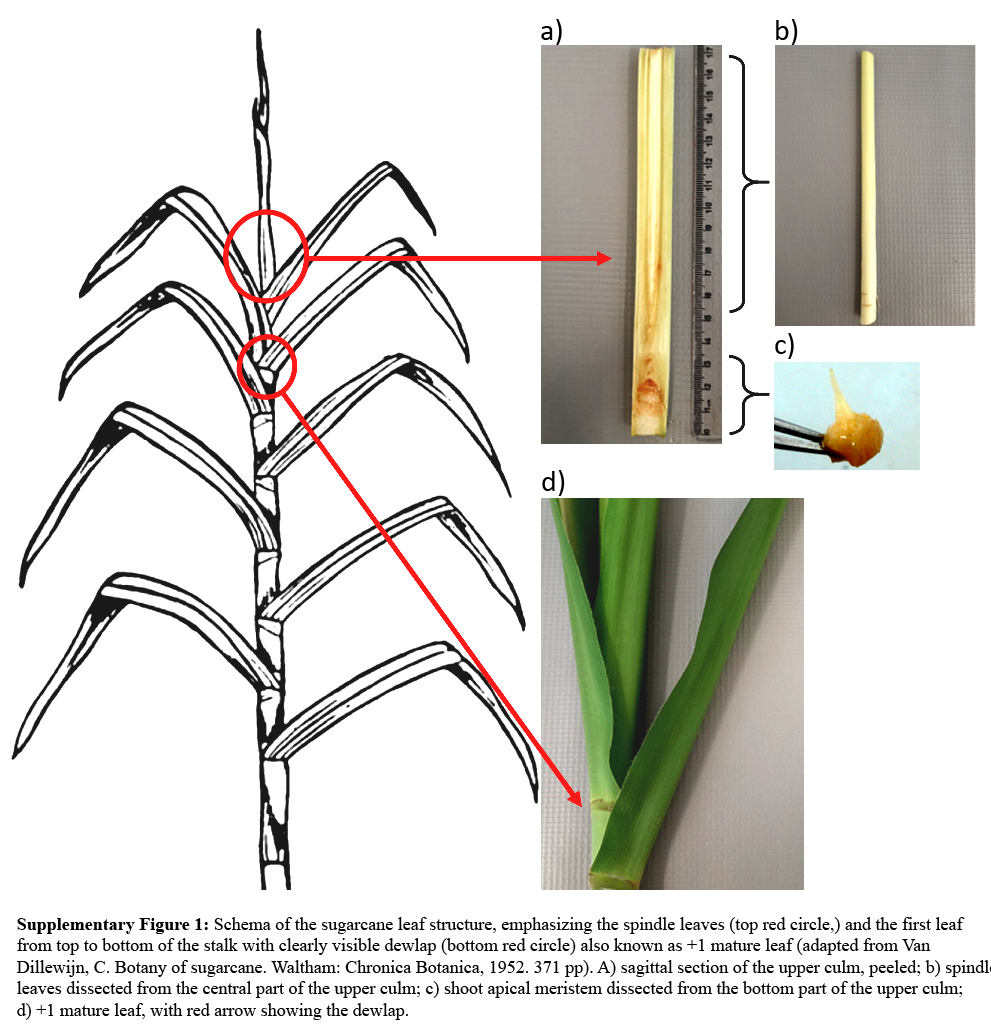

Supplement: Supplementary Figure 1 — Schema of the sugarcane leaf structure, emphasizing the spindle leaves (top red circle), and the first leaf from the top to the bottom of the stalk with clearly visible dewlap (bottom red circle) also known as +1 mature leaf (adapted from Van Dillewijn, C. Botany of sugarcane. Waltham: Chronica Botanica, 1952. 371). (A) sagittal section of the upper culm: peeled, (B) spindle leaves dissected from the central part of the upper culm, (C) shoot apical meristem dissected from the bottom part of the upper culm, and (D) +1 mature leaf with red arrow showing the dewlap. [file Image_1.tiff]

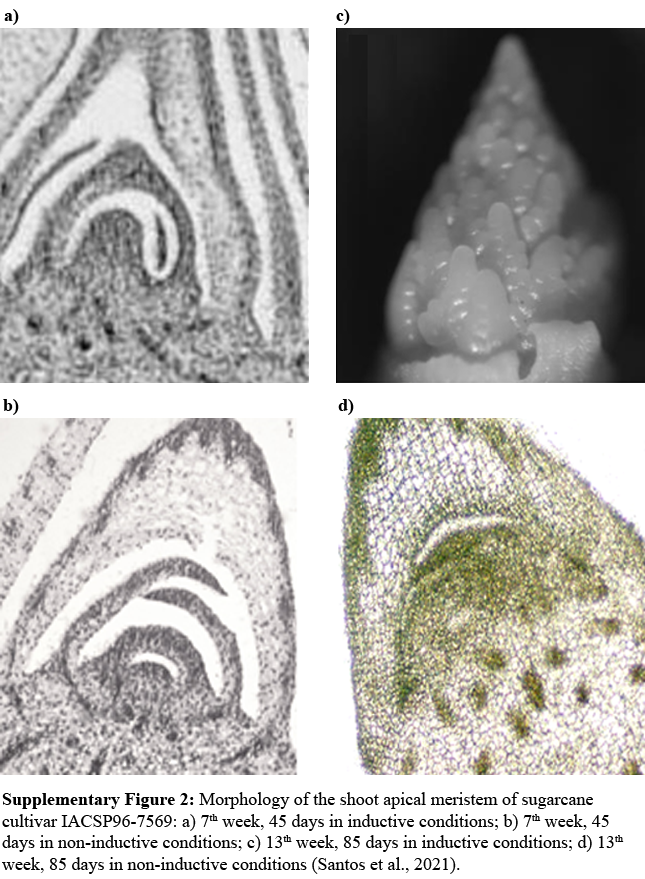

Supplement: Supplementary Figure 2 — Morphology of the shoot apical meristem of sugarcane cultivar IACSP96-7569: (A) seventh week, 45 days in inductive conditions; (B) seventh week, 45 days in non-inductive conditions; (C) 13th week, 85 days in inductive conditions; and (D) 13th week, 85 days in non-inductive conditions (Santos et al., 2021). [file Image_2.tiff]

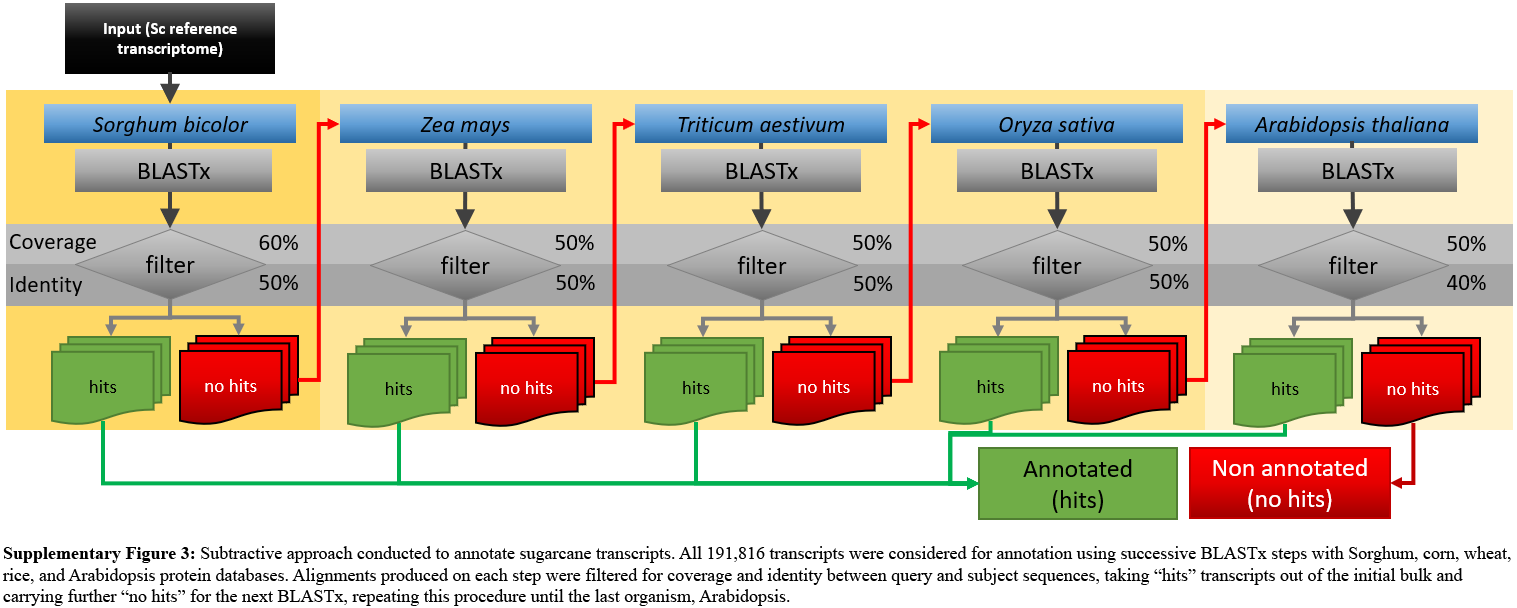

Supplement: Supplementary Figure 3 — Subtractive approach conducted to annotate sugarcane transcripts. All 191,816 transcripts were considered for annotation using successive BLASTx steps with sorghum, corn, wheat, rice, and Arabidopsis protein databases. Alignments produced in each step were filtered for coverage and identity between query and subject sequences, taking “hit” transcripts out of the initial bulk and carrying further “no hits” for the next BLASTx and repeating this procedure until the last organism, Arabidopsis. [file Image_3.TIF]

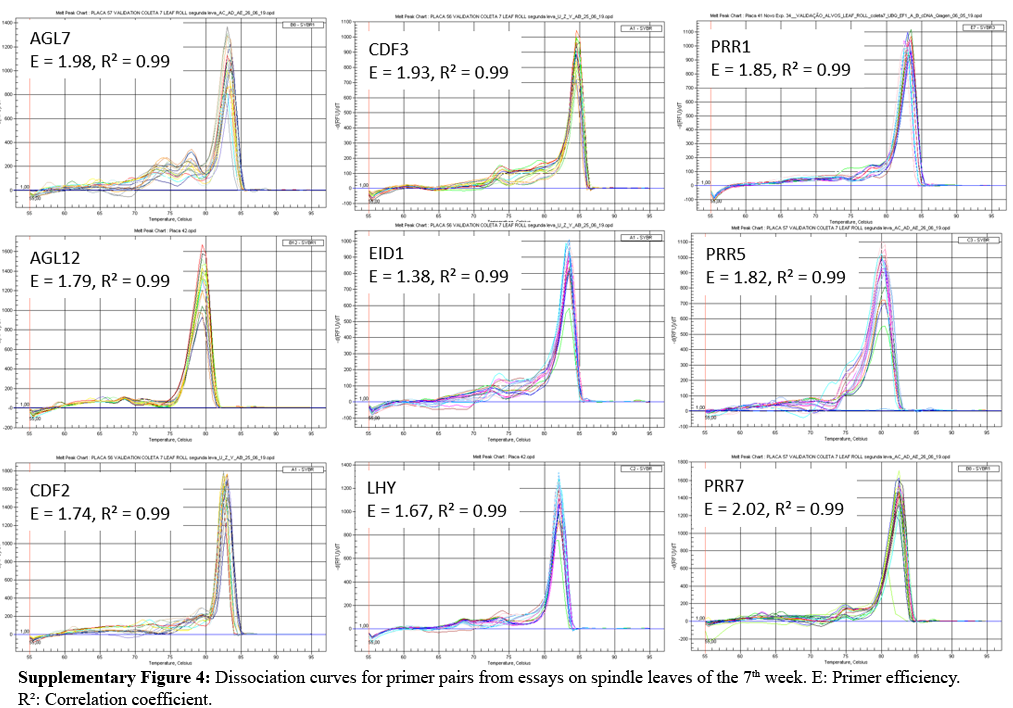

Supplement: Supplementary Figure 4 — Dissociation curves for primer pairs from essays on spindle leaves of the seventh week. E, primer efficiency; R2, Correlation coefficient. [file Image_4.TIF]

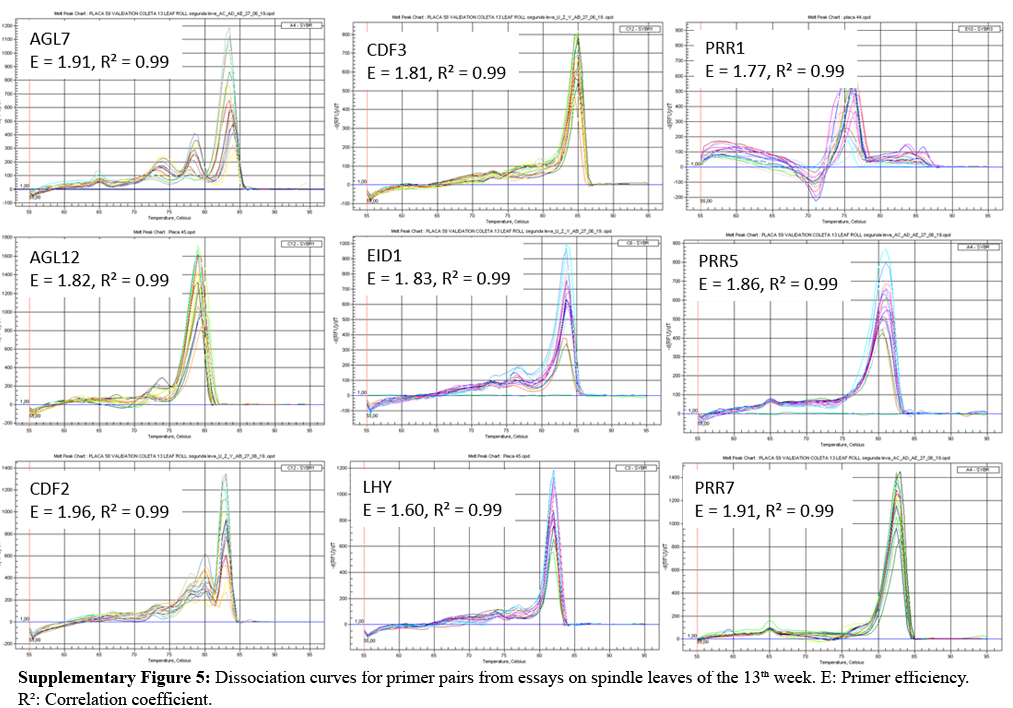

Supplement: Supplementary Figure 5 — Dissociation curves for primer pairs from essays on spindle leaves of the 13th week. E, primer efficiency; R2, correlation coefficient. [file Image_5.TIF]

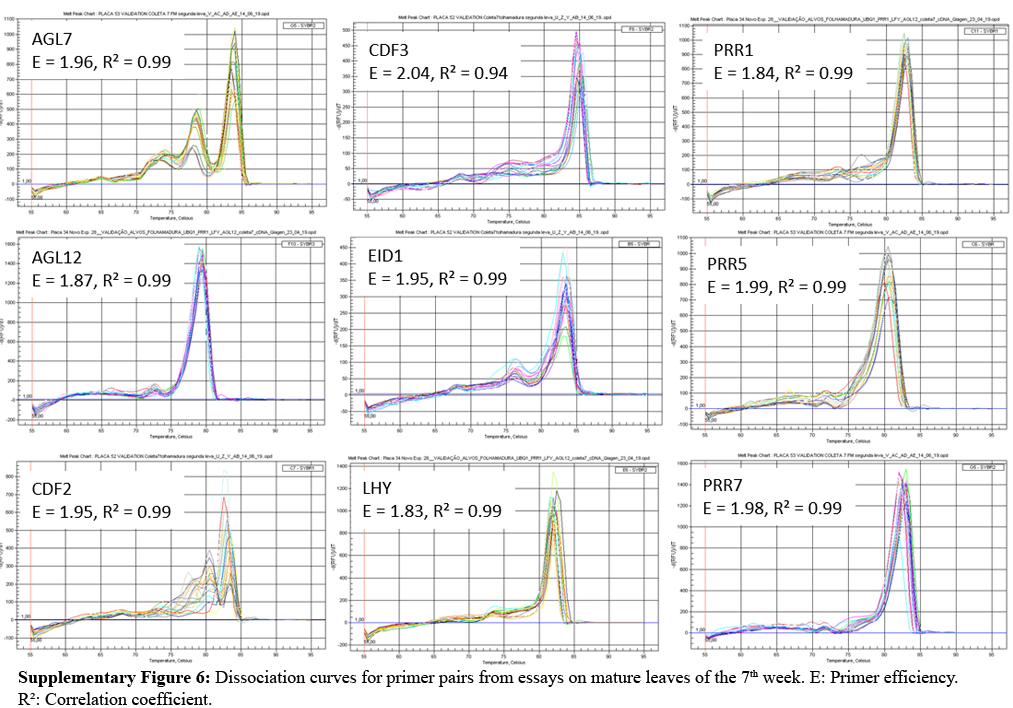

Supplement: Supplementary Figure 6 — Dissociation curves for primer pairs from essays on mature leaves of the seventh week. E, primer efficiency; R2, correlation coefficient. [file Image_6.TIF]

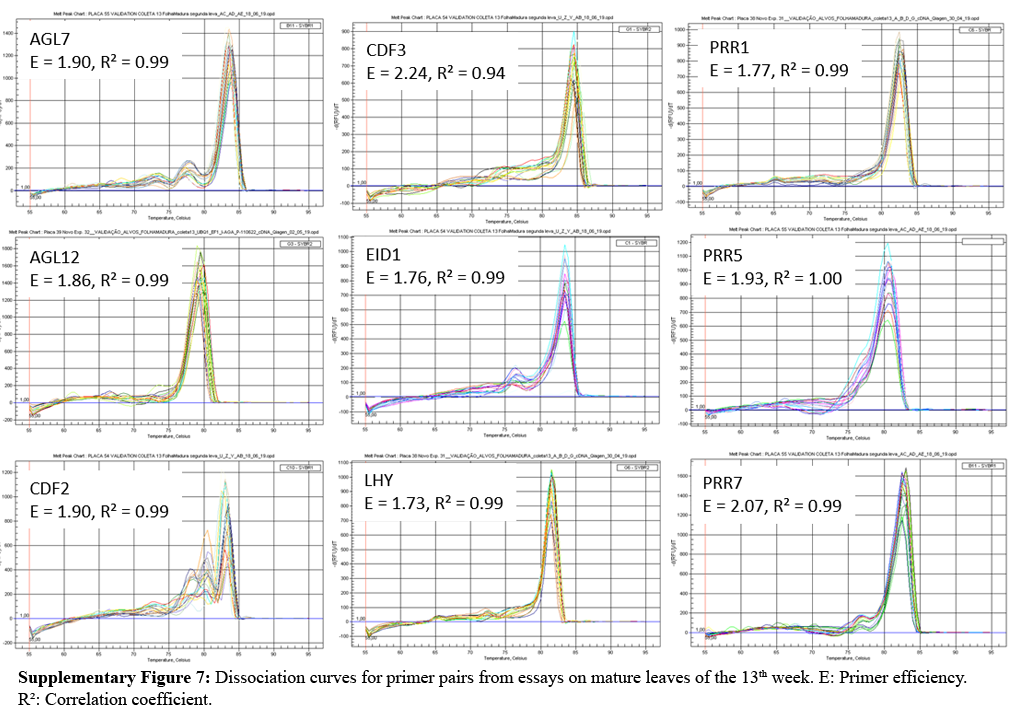

Supplement: Supplementary Figure 7 — Dissociation curves for primer pairs from essays on mature leaves of the 13th week. E, primer efficiency; R2, correlation coefficient. [file Image_7.TIF]

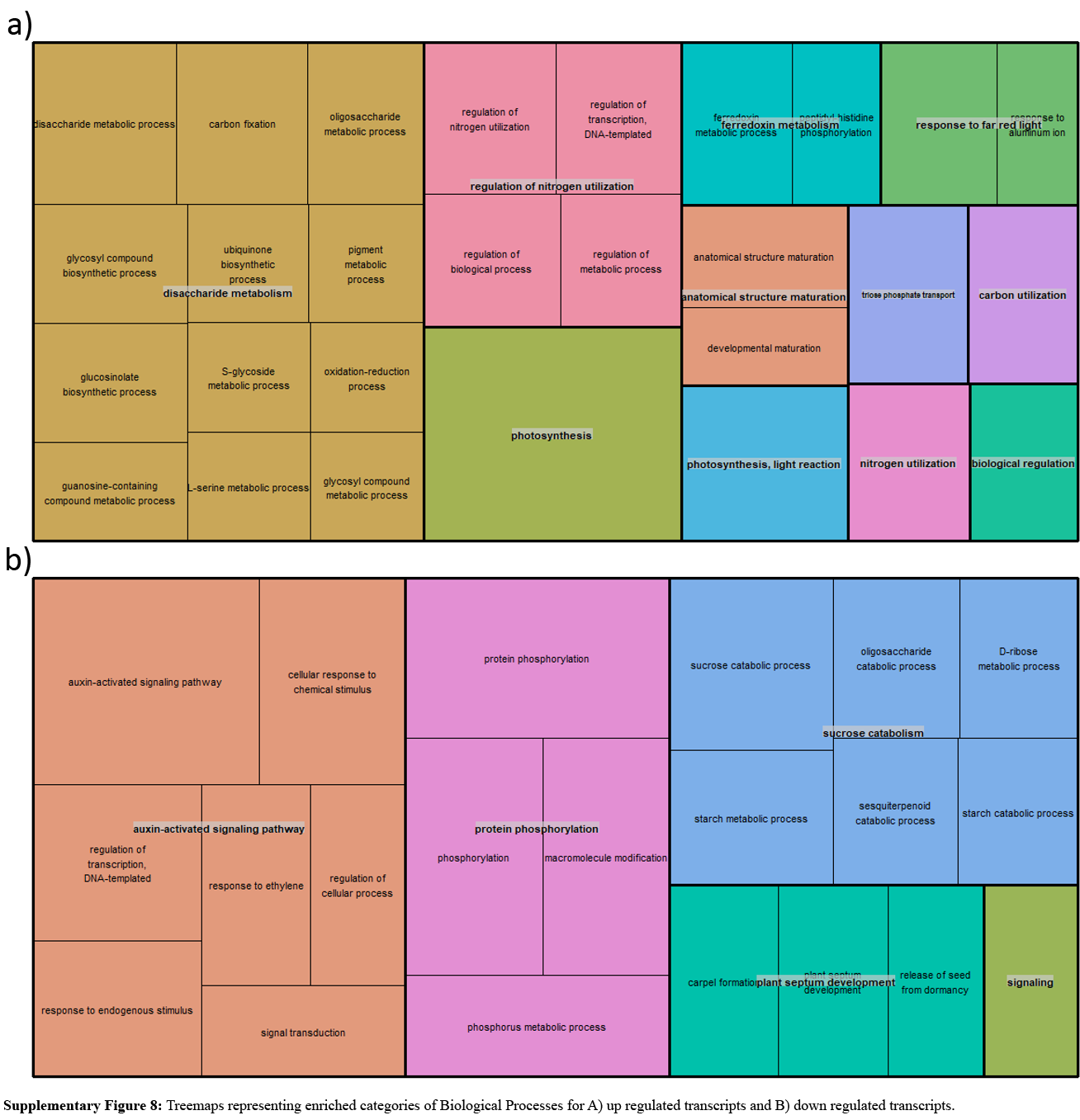

Supplement: Supplementary Figure 8 — Tree maps representing enriched categories of biological processes for (A) upregulated transcripts and (B) downregulated transcripts. [file Image_8.TIF]
