# Supplementary material for: Transcriptomic Analysis of Changes in Gene Expression During Flowering Induction in Sugarcane Under Controlled Photoperiodic Conditions
Source: Front Plant Sci. 2021 Jun 15;12:635784. doi: 10.3389/fpls.2021.635784 (PMC8239368; doi:10.3389/fpls.2021.635784)
Supplement: Supplementary Table 1 — Target sequences. Gene names (and Arabidopsis thaliana TAIR9 entry) used for orthology inference. [file Table_1.pdf]

Supplementary Table 1: Target sequences. Gene names (and *Arabidopsis thaliana* TAIR9 entry) used for orthology inference.

AP1 (AT1G69120)	CK2A3 (AT2G23080)	CRY2 (AT1G04400)	PHYB (AT2G18790)
AP2 (AT4G36920)	CK2A (AT2G23070)	ELF3 (AT2G25930)	PIF3 (AT1G09530)
ATC (AT2G27550)	CK2B1 (AT5G47080)	FKF1 (AT1G68050)	PRR3 (AT5G60100)
CCA1 (AT2G46830)	CK2B2 (AT4G17640)	FPF1 (AT5G24860)	PRR5 (AT5G24470)
CDF1 (AT5G62430)	CK2B3 (AT3G60250)	FT (AT1G65480)	PRR7 (AT5G02810)
CDF2 (AT5G39660)	CK2B4 (AT2G44680)	GIGANTEA (AT1G22770)	PRR9 (AT2G46790)
CDF3 (AT3G47500)	CKA1 (AT5G67380)	LFY (AT5G61850)	SOC1_AGL20 (AT2G45660)
CDF5 (AT1G69570)	CONSTANS (AT5G15840)	LHY (AT1G01060)	TLF1 (AT5G03840)
CHE (AT5G08330)	COP1 (AT2G32950)	PFT1 (AT1G25540)	TOC1 (AT5G61380)
CK2A2 (AT3G50000)	CRY1 (AT4G08920)	PHYA (AT1G09570)	ZTL (AT5G57360)
