# Supplementary material for: Transcriptomic Analysis of Changes in Gene Expression During Flowering Induction in Sugarcane Under Controlled Photoperiodic Conditions
Source: Front Plant Sci. 2021 Jun 15;12:635784. doi: 10.3389/fpls.2021.635784 (PMC8239368; doi:10.3389/fpls.2021.635784)
Supplement: Supplementary Table 2 — Main results and metrics of the reference-based mapping of RNA-Seq transcripts. [file Table_2.PDF]

Supplementary Table 2: Main results and metrics of the reference-based mapping of RNA-Seq transcripts.

**Atribute**	**Amount**	**Atribute**	**Amount**
Total ref. sequences	191,816	Sequences with ORF	63,028
Mapped sequences	112,584	% mean of ORFs	56.03%
Mean length	914	n50	1,815
Sequences < 200 bases	26,826	GC content	0.489
Sequences > 1000 bases	61,129		
