# Supplementary material for: Transcriptomic Analysis of Changes in Gene Expression During Flowering Induction in Sugarcane Under Controlled Photoperiodic Conditions
Source: Front Plant Sci. 2021 Jun 15;12:635784. doi: 10.3389/fpls.2021.635784 (PMC8239368; doi:10.3389/fpls.2021.635784)
Supplement: Supplementary Table 3 — Complete list with results from the phylogenetic analysis. Sugarcane transcripts were clustered closely to respective target A. thaliana sequences with correspondence to S. bicolor and O. sativa homologous genes. [file Table_3.PDF]

Supplementary Table 3: Complete list with results from the Phylexpress analysis. Sugarcane transcripts were clustered closely to respective target *A. thaliana* sequences, with correspondence to *S. bicolor* and *O. sativa* homologous genes.

Sugarcane transcript	Gene	Target sequences (*A. thaliana*)	Target sequence labels	*S. bicolor* homolog	*O. sativa* homolog	Putative *A. thaliana* to Sugarcane Ortholog
transcript_107397	AP2	A4G36920	AP2	Sb02g007000	Os07g13170	AtAP2, SbAP2, OsAP2
transcript_137721	ATC	A2G27550, A5G03840, A1G65480	ATC (TFL1, FT)	Sb08g003210	Os11g05470	AtCEN, SbATC, RCN1 (RICE CENTRORADIALIS1)
transcript_137722	ATC	A2G27550, A5G03840, A1G65480	ATC (TFL1, FT)	Sb08g003210	Os11g05470	AtCEN, SbATC, RCN1 (RICE CENTRORADIALIS1)
transcript_40257	CDF2	A5G39660, A5G62430, A3G47500, A1G69570	CDF2, CDF1, CDF3, CDF5	Sb03g011160	Os01g17000	AtCDF2, SbCDF2, OsDOF4
transcript_159837	CDF3	A3G47500, A5G62430, A5G39660, A1G69570	CDF3, CDF1, CDF2, CDF5	Sb02g043310	Os07g48570	AtCDF3, SbCDF2, OsDOF23
transcript_147392	CHE	A5G08330	CHE	Sb04g038140	Os04g11830	AtCHE, SbTCP, OsTCP15
transcript_147394	CHE	A5G08330	CHE	Sb04g038140	Os04g44440	AtCHE, SbTCP, OsTCP17
transcript_147395	CHE	A5G08330	CHE	Sb04g038140	Os02g42380	AtCHE, SbTCP, OsTCP7
transcript_111942	CKA3	A2G23080, A2G23070, A3G50000, A5G67380	CKA3, CKA4, CKA2, CKA1	Sb02g001110	Os03g55490	AtCKA3, SbCKA1, OsCKA2
transcript_102382	CKA3	A2G23080, A3G50000, A5G67380, A2G23070	CKA3, CKA2, CKA1, CKA4	Sb01g007270	Os07g02350	AtCKA3, SbCKA1, OsCKA2
transcript_477	CKA4	A2G23070	CKA4	Sb01g007190	Os03g55490	AtCKA4, SbCKA4
transcript_143148	CKA4	A2G23070	CKA4	Sb01g007190	Os03g55490	AtCKA4, SbCKA4
transcript_140728	CKB2	A4G17640	CKB2	Sb02g033960	Os07g31280	AtCKB2, SbCKB4
transcript_140729	CKB2	A4G17640	CKB2	Sb02g033960	Os07g31280	AtCKB2, SbCKB4
transcript_169418	CKB2	A4G17640, A5G47080, A2G44680, A3G60250	CKB2, CKB1, CKB4, CKB3	Sb06g024080	Os07g31280	AtCBK2, SbCKB1
transcript_6448	CKB3	A3G60250, A2G44680, A5G47080, A4G17640	CKB3, CKB4, CKB1, CKB2	Sb06g024080	Os10g41520	AtCKB3, SbCKB1
transcript_186685	CKB3	A3G60250, A2G44680, A5G47080, A4G17640	CKB3, CKB4, CKB1, CKB2	Sb01g028740	Os10g41520	AtCKB3, SbCKB4
transcript_186686	CKB3	A3G60250, A2G44680, A5G47080, A4G17640	CKB3, CKB4, CKB1, CKB2	Sb01g028740	Os10g41520	AtCKB3, SbCKB4
transcript_100622	CKB4	A2G44680, A5G47080	CKB4, CKB1	Sb06g024080	Os10g41520	AtCKB4, SbCKB1, OsCKB4
transcript_137173	COP1	A2G32950	COP1	Sb04g034480	Os02g53140	AtCOP1, SbCOP1
transcript_137174	COP1	A2G32950	COP1	Sb04g034480	Os02g53140	AtCOP1, SbCOP1
transcript_137175	COP1	A2G32950	COP1	Sb04g034480	Os02g53140	AtCOP1, SbCOP1
transcript_137176	COP1	A2G32950	COP1	Sb04g034480	Os02g53140	AtCOP1, SbCOP1
transcript_100230	CRY1	A4G08920, A1G04400	CRY1, CRY2	Sb06g018510	Os4g37920	AtCRY1, SbCRY1
transcript_117620	CRY1	A4G08920, A1G04400	CRY1, CRY2	Sb04g023680	Os02g36380	AtCRY1, SbCRY1
transcript_117621	CRY1	A4G08920, A1G04400	CRY1, CRY2	Sb04g023680	Os02g36380	AtCRY1, SbCRY1
transcript_117622	CRY1	A4G08920, A1G04400	CRY1, CRY2	Sb04g023680	Os02g36380	AtCRY1, SbCRY1
transcript_169851	CRY2	A1G04400, A4G08920	CRY2, CRY1	Sb10g013750	Os02g41550	AtCRY2, SbCRY2
transcript_169852	CRY2	A1G04400, A4G08920	CRY2, CRY1	Sb10g013750	Os02g41550	AtCRY2, SbCRY2
transcript_169853	CRY2	A1G04400, A4G08920	CRY2, CRY1	Sb10g013750	Os02g41550	AtCRY2, SbCRY2
transcript_169855	CRY2	A1G04400, A4G08920	CRY2, CRY1	Sb10g013750	Os02g41550	AtCRY2, SbCRY2
transcript_117672	FKF1	A1G68050, A5G57360	FKF1, ZTL	Sb05g021030	Os11g34460	AtFKF1, SbFKF1, OsFKF1
transcript_157360	FT	A1G65480	FT	Sb03g001700	Os01g11940	AtFT, SbFT, OsFTL1
transcript_157361	FT	A1G65480	FT	Sb03g001700	Os01g11940	AtFT, SbFT, OsFTL1
transcript_157362	FT	A1G65480	FT	Sb03g001700	Os01g11940	AtFT, SbFT, OsFTL1
transcript_148722	FT	A1G65480, A2G27550, A5G03840	FT, ATC, TFL1	Sb06g020850	Os04g41130	AtFT, SbFTL6, OsFTL6
transcript_110985	FT	A1G65480, A2G27550	FT, ATC	Sb03g002500	Os01g10590	AtBFT, SbTSF, OsFTL8
transcript_158433	GI	A1G22770	GI	Sb03g003650	Os01g08700	AtGI, SbGI, OsGI
transcript_158434	GI	A1G22770	GI	Sb03g003650	Os01g08700	AtGI, SbGI, OsGI
transcript_158435	GI	A1G22770	GI	Sb03g003650	Os01g08700	AtGI, SbGI, OsGI
transcript_158436	GI	A1G22770	GI	Sb03g003650	Os01g08700	AtGI, SbGI, OsGI
transcript_158437	GI	A1G22770	GI	Sb03g003650	Os01g08700	AtGI, SbGI, OsGI
transcript_137919	LHY1, CCA1	A1G01060, A2G46830	LHY1, CCA1	Sb07g003870	Os08g06110	AtLHY, SbLHY, OsLHY
transcript_137920	LHY1, CCA1	A1G01060, A2G46830	LHY1, CCA1	Sb07g003870	Os08g06110	AtLHY, SbLHY, OsLHY
transcript_137921	LHY1, CCA1	A1G01060, A2G46830	LHY1, CCA1	Sb07g003870	Os08g06110	AtLHY, SbLHY, OsLHY
transcript_137922	LHY1, CCA1	A1G01060, A2G46830	LHY1, CCA1	Sb07g003870	Os08g06110	AtLHY, SbLHY, OsLHY
transcript_140431	PFT1	A1G25540	PFT1	Sb02g020790	Os09g13610	AtPFT1, SbPFT1, OsPFT1
transcript_140435	PFT1	A1G25540	PFT1	Sb02g020790	Os09g13610	AtPFT1, SbPFT1, OsPFT1

transcript_140436	PFT1	A1G25540	PFT1	Sb02g020790	Os09g13610	AtPFT1, SbPFT1, OsPFT1
transcript_140437	PFT1	A1G25540	PFT1	Sb02g020790	Os09g13610	AtPFT1, SbPFT1, OsPFT1
transcript_140438	PFT1	A1G25540	PFT1	Sb02g020790	Os09g13610	AtPFT1, SbPFT1, OsPFT1
transcript_160479	PHYa, PHYb	A1G09570, A2G18790	PHYa, PHYb	Sb01g009920	Os03g51030	AtPHYa, SbPHYa, OsPHYa
transcript_160480	PHYa, PHYb	A1G09570, A2G18790	PHYa, PHYb	Sb01g009920	Os03g51030	AtPHYa, SbPHYa, OsPHYa
transcript_129084	PHYa, PHYb	A1G09570, A2G18790	PHYa, PHYb	Sb01g007850	Os03g54084	AtPHYa, SbPHYc, OsPHYc
transcript_129085	PHYa, PHYb	A1G09570, A2G18790	PHYa, PHYb	Sb01g007850	Os03g54084	AtPHYa, SbPHYc, OsPHYc
transcript_151669	PRR5, PRR9, PRR3, PRR7, TOC1	A5G24470, A5G02810, A2G46790, A5G60100, A5G61380	PRR5, PRR9, PRR3, PRR7, TOC1	Sb05g003660	Os11g05930	AtPRR5, SbPRR9, OsPRR59
transcript_151675	PRR5, PRR9, PRR3, PRR7, TOC1	A5G02810, A5G24470, A2G46790, A5G60100, A5G61380	PRR5, PRR9, PRR3, PRR7, TOC1	Sb05g003660	Os11g05930	AtPRR7, SbPRR9, OsPRR59
transcript_129241	PRR7, PRR3, PRR5, PRR9, TOC1	A5G02810, A5G60100, A5G24470, A2G46790, A5G61380	PRR7, PRR3, PRR5, PRR9, TOC1	Sb01g038820	Os03g17570	AtPRR7, SbPRR7, OsPRR73
transcript_129242	PRR7, PRR3, PRR5, PRR9, TOC1	A5G02810, A5G60100, A5G24470, A2G46790, A5G61380	PRR7, PRR3, PRR5, PRR9, TOC1	Sb01g038820	Os03g17570	AtPRR7, SbPRR7, OsPRR73
transcript_129243	PRR7, PRR3, PRR5, PRR9, TOC1	A5G02810, A5G60100, A5G24470, A2G46790, A5G61380	PRR7, PRR3, PRR5, PRR9, TOC1	Sb01g038820	Os03g17570	AtPRR7, SbPRR7, OsPRR73
transcript_133513	PRR7, PRR3, PRR5, PRR9, TOC1	A5G02810, A5G60100, A5G24470, A2G46790, A5G61380	PRR7, PRR3, PRR5, PRR9, TOC1	Sb06g014570	Os07g49460	AtPRR7, SbPRR3, OsPRR37
transcript_151673	PRR7, PRR3, PRR9, PRR5, TOC1	A5G02810, A5G60100, A2G46790, A5G24470, A5G61380	PRR7, PRR3, PRR9, PRR5, TOC1	Sb05g003660	Os11g05930	AtPRR7, SbPRR9, OsPRR59
transcript_151674	PRR7, PRR3, PRR9, PRR5, TOC1	A5G02810, A5G60100, A2G46790, A5G24470, A5G61380	PRR7, PRR3, PRR9, PRR5, TOC1	Sb05g003660	Os11g05930	AtPRR7, SbPRR9, OsPRR59
transcript_173081	SOC1	A2G45660	SOC1	Sb01g049020	Os03g03070	OsSOC1, SbSOC1, AtSOC1
transcript_173082	SOC1	A2G45660	SOC1	Sb01g049020	Os03g03070	OsSOC1, SbSOC1, AtSOC1
transcript_173083	SOC1	A2G45660	SOC1	Sb01g049020	Os03g03070	OsSOC1, SbSOC1, AtSOC1
transcript_154344	SOC1	A2G45660	SOC1	Sb01g030570	Os10g39130	AtSOC1, SbAGL20, OsMADS56
transcript_154345	SOC1	A2G45660	SOC1	Sb01g030570	Os10g39130	AtSOC1, SbAGL20, OsMADS56
transcript_165795	TOC1, PRR9, PRR7, PRR3, PRR5	A5G61380, A2G46790, A5G02810, A5G60100, A5G24470	TOC1, PRR9, PRR7, PRR3, PRR5	Sb04g026190	Os02g40510	AtTOC1, SbTOC1, OsPRR1
transcript_101349	ZTL, FKF1	A5G57360, A1G68050	ZTL, FKF1	Sb10g028340	Os06g47890	AtZTL, SbZTL, OsFBO9
transcript_153107	ZTL, FKF1	A5G57360, A1G68050	ZTL, FKF1	Sb04g003660	Os02g05700	AtZTL, SbZTL
transcript_153108	ZTL, FKF1	A5G57360, A1G68050	ZTL, FKF1	Sb04g003660	Os02g05700	AtZTL, SbZTL
transcript_153109	ZTL, FKF1	A5G57360, A1G68050	ZTL, FKF1	Sb04g003660	Os02g05700	AtZTL, SbZTL
transcript_153110	ZTL, FKF1	A5G57360, A1G68050	ZTL, FKF1	Sb04g003660	Os02g05700	AtZTL, SbZTL
transcript_153111	ZTL, FKF1	A5G57360, A1G68050	ZTL, FKF1	Sb04g003660	Os02g05700	AtZTL, SbZTL
transcript_153112	ZTL, FKF1	A5G57360, A1G68050	ZTL, FKF1	Sb04g003660	Os02g05700	AtZTL, SbZTL
