# Supplementary material for: Transcriptomic Analysis of Changes in Gene Expression During Flowering Induction in Sugarcane Under Controlled Photoperiodic Conditions
Source: Front Plant Sci. 2021 Jun 15;12:635784. doi: 10.3389/fpls.2021.635784 (PMC8239368; doi:10.3389/fpls.2021.635784)
Supplement: Supplementary Table 4 — Gene relative expression conduced with RT-qPCR essays for two time points (seventh week, 45 days of treatment and 13th week, 85 days of treatment) and two tissues (spindle leaves and mature leaves). [file Table_4.pdf]

Supplementary Table 4: Gene relative expression conduced with RT-qPCR essays for two time points (7th week, 45 days of treatment, and 13th week, 85 days of treatment) and two tissues (spindle leaves and mature leaves).

		7th Week								13th Week							
	Gene	Reaction Efficiency	Relative Expression	Standard Error		95% Confidence Interval		P(H1)	Result	Reaction Efficiency	Relative Expression	Standard Error		95% Confidence Interval		P(H1)	Result
Spindle Leaf	ScAGL7	0.90	0.45	0.21	0.97	0.09	1.64	0.01	REPRESSED*	0.90	1.94	0.95	3.91	0.61	7.77	0.01	INDUCED*
	ScAGL12	0.70	1.84	0.96	4.12	0.65	5.37	0.01	INDUCED*	0.80	1.98	0.96	3.97	0.82	12.06	0.01	INDUCED**
	ScCDF2	0.70	1.48	0.83	2.24	0.53	6.06	0.07	*NA*	0.90	2.89	1.54	5.77	1.10	12.11	0.00	INDUCED
	ScCDF3	0.90	0.57	0.34	1.00	0.27	1.48	0.01	REPRESSED*	0.90	2.80	1.34	5.99	0.61	12.70	0.00	INDUCED**
	ScEID1	0.30	0.82	0.55	1.13	0.43	2.16	0.20	*NA*	0.80	2.28	0.93	5.21	0.46	16.73	0.02	INDUCED*
	ScLHY	0.60	1.20	0.74	2.04	0.50	3.16	0.31	*NA*	0.60	2.57	1.24	6.09	0.65	12.79	0.00	INDUCED
	ScPRR1	0.80	0.70	0.43	1.13	0.24	1.74	0.07	*NA*	0.70	4.67	2.15	10.12	1.14	18.40	0.00	INDUCED
	ScPRR5	0.80	0.89	0.59	1.39	0.41	1.89	0.41	*NA*	0.80	1.83	0.90	5.11	0.53	7.35	0.04	INDUCED*
	ScPRR7	1.00	1.50	0.65	5.49	0.12	9.68	0.32	*NA*	0.90	4.94	2.28	13.77	1.27	25.34	0.00	INDUCED
	ScUBQ1[1]	0.99	0.98	*NA*	*NA*	*NA*	*NA*	*NA*	*NA*	0.99	0.59	*NA*	*NA*	*NA*	*NA*	*NA*	*NA*
	ScTUB[1]	1.00	1.03	*NA*	*NA*	*NA*	*NA*	*NA*	*NA*	1.00	1.71	*NA*	*NA*	*NA*	*NA*	*NA*	*NA*
Mature Leaf	ScAGL7	0.90	1.22	0.83	1.94	0.55	2.65	0.20	*NA*	0.90	1.29	0.90	1.80	0.58	2.16	0.06	*NA*
	ScAGL12	0.80	1.15	0.67	2.09	0.36	2.44	0.48	*NA*	0.80	1.93	1.02	4.53	0.70	6.38	0.01	INDUCED*
	ScCDF2	0.90	2.44	1.15	5.28	0.68	8.82	0.00	INDUCED***	0.90	1.14	0.55	2.27	0.44	3.36	0.54	*NA*
	ScCDF3	1.00	1.76	1.26	2.45	0.98	2.84	0.00	INDUCED	1.00	0.79	0.16	4.07	0.10	21.52	0.65	*NA*
	ScEID1	0.90	1.22	0.80	1.79	0.54	2.36	0.16	*NA*	0.70	0.80	0.26	1.98	0.15	4.71	0.52	*NA*
	ScLHY	0.80	1.47	0.81	2.72	0.49	3.80	0.08	*NA*	0.70	0.80	0.39	1.71	0.13	2.62	0.50	*NA*
	ScPRR1	0.80	0.52	0.35	0.70	0.25	1.03	0.00	REPRESSED	0.80	1.03	0.60	1.57	0.36	3.03	0.86	*NA*
	ScPRR5	0.90	1.20	0.55	3.22	0.43	10.25	0.62	*NA*	0.90	1.57	0.85	2.49	0.57	4.37	0.04	INDUCED*
	ScPRR7	0.90	1.55	0.78	3.22	0.49	5.95	0.09	*NA*	1.00	1.91	0.97	3.76	0.55	8.37	0.03	INDUCED*
	ScUBQ1[1]	0.90	1.23	*NA*	*NA*	*NA*	*NA*	*NA*	*NA*	0.93	1.25	*NA*	*NA*	*NA*	*NA*	*NA*	*NA*
	ScTUB[1]	1.10	0.81	*NA*	*NA*	*NA*	*NA*	*NA*	*NA*	1.10	0.80	*NA*	*NA*	*NA*	*NA*	*NA*	*NA*

[1]Reference genes. P(H1) Null hypothesys: P <0.001 (***), P<0.01(**), P< 0.05(*).
