# Supplementary material for: Transcriptomic Analysis of Changes in Gene Expression During Flowering Induction in Sugarcane Under Controlled Photoperiodic Conditions
Source: Front Plant Sci. 2021 Jun 15;12:635784. doi: 10.3389/fpls.2021.635784 (PMC8239368; doi:10.3389/fpls.2021.635784)
Supplement: Supplementary Table 5 — List of overrepresented enriched GO terms (FDR < 0.05) from sugarcane photoperiodic flowering induction transcriptome. [file Table_5.PDF]

Supplementary Table 5: List of overrepresented enriched GO terms (FDR < 0.05) for Biological Processes from sugarcane photoperiodic flowering induction transcriptome.

DE	GO ID	GO Name	GO Cat.	FDR	P-Value	N#
UP	GO:0006355	regulation of transcription, DNA-templated	BP	0.0026	1.43E-05	79
UP	GO:0042440	pigment metabolic process	BP	0.0260	3.31E-04	12
UP	GO:0006808	regulation of nitrogen utilization	BP	0.0019	7.71E-06	8
UP	GO:0009765	photosynthesis, light harvesting	BP	0.0011	3.67E-06	8
UP	GO:0005985	sucrose metabolic process	BP	0.0026	1.41E-05	7
UP	GO:0006563	L-serine metabolic process	BP	0.0293	3.89E-04	6
UP	GO:0018106	peptidyl-histidine phosphorylation	BP	0.0186	2.21E-04	6
UP	GO:1901068	guanosine-containing compound metabolic process	BP	0.0117	1.27E-04	6
UP	GO:0010218	response to far red light	BP	0.0026	1.48E-05	6
UP	GO:0048765	root hair cell differentiation	BP	0.0448	6.60E-04	5
UP	GO:0019253	reductive pentose-phosphate cycle	BP	0.0065	6.38E-05	5
UP	GO:0010114	response to red light	BP	0.0035	2.70E-05	5
UP	GO:0009773	photosynthetic electron transport in photosystem I	BP	0.0364	5.08E-04	4
UP	GO:0010044	response to aluminum ion	BP	0.0323	4.40E-04	4
UP	GO:0006744	ubiquinone biosynthetic process	BP	0.0194	2.32E-04	4
UP	GO:0019761	glucosinolate biosynthetic process	BP	0.0029	2.08E-05	4
UP	GO:0015976	carbon utilization	BP	0.0022	9.81E-06	4
UP	GO:0015717	triose phosphate transport	BP	0.0011	3.83E-06	4
UP	GO:0019464	glycine decarboxylation via glycine cleavage system	BP	0.0431	6.20E-04	3
UP	GO:0006124	ferredoxin metabolic process	BP	0.0032	2.35E-05	3
DOWN	GO:0006468	protein phosphorylation	BP	0.0000	1.30E-08	96
DOWN	GO:0006355	regulation of transcription, DNA-templated	BP	0.0032	1.13E-05	69
DOWN	GO:0009734	auxin-activated signaling pathway	BP	0.0000	1.85E-09	17
DOWN	GO:0009723	response to ethylene	BP	0.0134	8.30E-05	8
DOWN	GO:0006014	D-ribose metabolic process	BP	0.0370	2.95E-04	4
DOWN	GO:0005983	starch catabolic process	BP	0.0481	4.90E-04	4
DOWN	GO:0005987	sucrose catabolic process	BP	0.0019	5.52E-06	3
DOWN	GO:0010500	transmitting tissue development	BP	0.0182	1.25E-04	2
DOWN	GO:0048462	carpel formation	BP	0.0182	1.25E-04	2
DOWN	GO:0080126	ovary septum development	BP	0.0182	1.25E-04	2
DOWN	GO:0048838	release of seed from dormancy	BP	0.0400	3.72E-04	2
DOWN	GO:0046345	abscisic acid catabolic process	BP	0.0400	3.72E-04	2
