# Supplementary material for: Transcriptomic Analysis of Changes in Gene Expression During Flowering Induction in Sugarcane Under Controlled Photoperiodic Conditions
Source: Front Plant Sci. 2021 Jun 15;12:635784. doi: 10.3389/fpls.2021.635784 (PMC8239368; doi:10.3389/fpls.2021.635784)
Supplement: Supplementary Table 6 — List of conserved sugarcane lncRNAs responsive to flowering induction and its orthologs in S. bicolor, Z. mays, and S. italica (sorted by highest to lowest log2 fold change). [file Table_6.PDF]

Supplementary Table 6: List of conserved sugarcane lncRNAs responsive to flowering induction and its orthologs in *S. bicolor* , *Z. mays* , and *S. italica* (sorted by highest to lowest log2 Fold Change).

Organism	Sc transcript	Locus name	log2 Fold Change	lfcSE	pvalue	padj	Defline
S. bicolor	transcript_141712	Sobic.007G034300	5,970518835	1,396096121	1,90E-05	5,34E-04	weakly similar to Putative uncharacterized protein OJ1119_D01.15
	transcript_110367	Sobic.008G091100	5,695452367	1,265116511	6,73E-06	2,22E-04	NA
	transcript_141708	Sobic.002G048900; Sobic.002G074600; Sobic.002G129900; Sobic.003G287450; Sobic.003G352750; Sobic.006G113100; Sobic.009G209650; Sobic.009G253900	3,819378194	0,624391824	9,54E-10	7,98E-08	weakly similar to Putative uncharacterized protein
	transcript_61088	Sobic.001G100501	3,027686836	0,439077163	5,36E-12	6,78E-10	weakly similar to Expressed protein
	transcript_141711	Sobic.007G034300	2,805147635	0,75858837	2,17E-04	0,004121725	weakly similar to Putative uncharacterized protein OJ1119_D01.15
	transcript_141710	Sobic.007G034300	2,520370265	0,357673103	1,83E-12	2,52E-10	weakly similar to Putative uncharacterized protein OJ1119_D01.15
	transcript_141709	Sobic.007G034300	2,355013828	0,53440202	1,05E-05	3,21E-04	weakly similar to Putative uncharacterized protein OJ1119_D01.15
	transcript_167515	Sobic.002G167250	1,941471219	0,529057643	2,43E-04	0,004514981	NA
	transcript_141706	Sobic.002G048900; Sobic.002G074600; Sobic.002G129900; Sobic.003G287450; Sobic.003G352750; Sobic.006G113100; Sobic.007G034300; Sobic.007G163001; Sobic.008G159200; Sobic.009G209650; Sobic.009G253900	1,797992321	0,297669184	1,54E-09	1,23E-07	weakly similar to Putative uncharacterized protein; weakly similar to Putative uncharacterized protein OJ1119_D01.15
	transcript_161477	Sobic.002G086200	1,790276733	0,534333406	8,07E-04	0,011852997	NA
	transcript_159982	Sobic.001G116650; Sobic.004G174500; Sobic.007G025350; Sobic.007G085350; Sobic.010G263950	1,512914677	0,328794388	4,20E-06	1,49E-04	NA
	transcript_186016	Sobic.003G175300	1,398475322	0,335471042	3,06E-05	8,06E-04	weakly similar to Putative uncharacterized protein
	transcript_161473	Sobic.003G175950	1,311499344	0,469829007	0,005247413	0,049482477	NA
	transcript_145688	Sobic.002G132500; Sobic.005G086650	1,293084254	0,308699793	2,80E-05	7,48E-04	NA
	transcript_111333	Sobic.001G250150; Sobic.001G251650; Sobic.001G291600; Sobic.003G404050; Sobic.004G007100; Sobic.006G029550; Sobic.009G217450	1,216992852	0,273657459	8,70E-06	2,75E-04	NA
	transcript_145689	Sobic.002G132500; Sobic.005G086650	1,130743618	0,18644154	1,32E-09	1,08E-07	NA
	transcript_132325	Sobic.006G160600; Sobic.006G210266; Sobic.007G059300; Sobic.008G106600	1,057200482	0,308441331	6,09E-04	0,009436029	similar to OSIGBa0159I10.14 protein
	transcript_120439	Sobic.005G137500; Sobic.K044402	1,021358909	0,295887609	5,57E-04	0,008802291	2.7.1.137 - Phosphatidylinositol 3-kinase / Type III phosphoinositide 3-kinase (1 of 4)
	transcript_102097	Sobic.009G064832	0,976906273	0,315973106	0,001989879	0,02374977	NA
	transcript_149	Sobic.006G025900	0,951939582	0,34102274	0,005247643	0,049482477	NA
	transcript_143049	Sobic.007G004850	0,812518242	0,147143782	3,35E-08	2,01E-06	NA
	transcript_142937	Sobic.003G176400	0,788052616	0,200807232	8,69E-05	0,001922244	NA
	transcript_107011	Sobic.003G173950; Sobic.008G093500	0,787027418	0,281298816	0,005144653	0,048795204	PF12353 - Eukaryotic translation initiation factor 3 subunit G (eIF3g) (1 of 4)
	transcript_23437	Sobic.002G035733	0,782408904	0,237546721	9,89E-04	0,013861183	NA

	transcript_113332	Sobic.004G108501	0,71706948	0,224649298	0,001413155	0,018299818	NA
	transcript_128304	Sobic.002G219366; Sobic.004G159866; Sobic.K044402	-0,627578782	0,207787366	0,0025253	0,028466284	NA
	transcript_135472	Sobic.001G245650	-0,632952044	0,189434546	8,34E-04	0,012164956	NA
	transcript_108195	Sobic.001G376150; Sobic.006G029550	-0,793391751	0,240770348	9,83E-04	0,013801114	NA
	transcript_106248	Sobic.005G096700; Sobic.005G096800	-0,800979568	0,1894513	2,36E-05	6,46E-04	NA
	transcript_66619	Sobic.010G214500	-0,831190435	0,188493979	1,04E-05	3,18E-04	3.1.3.8 - 3-phytase / Phytate 6-phosphatase (1 of 2)
	transcript_176716	Sobic.001G467150	-0,914789068	0,295954499	0,001994979	0,023802103	NA
	transcript_107726	Sobic.001G376150; Sobic.006G029550	-1,102741294	0,250683835	1,09E-05	3,31E-04	NA
	transcript_159494	Sobic.002G120850	-1,417639953	0,482093542	0,003275867	0,034722314	NA
	transcript_106236	Sobic.005G096700; Sobic.005G096800	-1,435224028	0,504622444	0,00445295	0,043679847	NA
	transcript_26059	Sobic.006G273100	-1,513948411	0,509087682	0,002940925	0,03195041	NA
	transcript_114139	Sobic.002G101600	-1,681680131	0,578437369	0,003645852	0,037596438	NA
	transcript_182864	Sobic.006G041200	-2,552969247	0,700805486	2,70E-04	0,004900884	NA
	transcript_106715	Sobic.005G086650	-3,193016325	1,052390228	0,002412855	0,027520271	NA
	transcript_156384	Sobic.001G059001	-3,195160629	1,035568649	0,002032673	0,024165211	NA
	transcript_106259	Sobic.005G096700; Sobic.005G096800; Sobic.007G047450	-3,230795294	0,841623783	1,24E-04	0,002572605	NA
	transcript_161304	Sobic.007G044350	-7,049865221	1,455684537	1,28E-06	5,32E-05	NA
Z. mays	transcript_141064	GRMZM2G410595	6,015555104	1,751610221	5,94E-04	0,009262596	NA
	transcript_146180	GRMZM2G080642	3,706156288	0,931661256	6,95E-05	0,001594839	NA
	transcript_14645	GRMZM2G178593	1,981678858	0,569884393	5,06E-04	0,008167452	PTHR10788:SF6 - BCDNA.GH08860 (1 of 5)
	transcript_9980	GRMZM2G079308	1,741154704	0,497664292	4,68E-04	0,007644355	K10781 - fatty acyl-ACP thioesterase B (FATB) (1 of 6). With phase in ZT20 in Light (12h)/Dark (12h)
	transcript_111346	GRMZM2G315279; GRMZM2G005747	1,601376901	0,518972396	0,002030949	0,024149038	NA
	transcript_284	GRMZM2G467997	1,594636101	0,507858376	0,001689924	0,020986133	PTHR10120//PTHR10120:SF21 - CAAX PRENYL PROTEASE 1 // SUBFAMILY NOT NAMED (1 of 6)
	transcript_15892	GRMZM2G010034	1,2696249	0,366693744	5,35E-04	0,008522253	NA
	transcript_173596	GRMZM2G143332	1,132647807	0,186346103	1,22E-09	1,00E-07	PTHR11913//PTHR11913:SF26 - COFILIN-RELATED // SUBFAMILY NOT NAMED (1 of 5)
	transcript_3464	GRMZM2G439660	1,118270605	0,398880488	0,005054763	0,048212159	NA
	transcript_144700	GRMZM2G027183	1,066794668	0,219416498	1,16E-06	4,92E-05	NA
	transcript_102097	GRMZM2G056068	0,976906273	0,315973106	0,001989879	0,02374977	PF12734 - Cysteine-rich TM module stress tolerance (CYSTM) (1 of 9)
	transcript_149487	GRMZM2G002756	0,920985908	0,265698214	5,28E-04	0,008433254	K06891 - ATP-dependent Clp protease adaptor protein ClpS (clpS) (1 of 2)
	transcript_126248	GRMZM2G118385	0,918004702	0,321644639	0,004315948	0,042669173	K10640 - E3 ubiquitin-protein ligase RNF25 [EC:6.3.2.19] (RNF25, AO7) (1 of 1)
	transcript_149486	GRMZM2G002756	0,890162175	0,184976698	1,49E-06	6,07E-05	K06891 - ATP-dependent Clp protease adaptor protein ClpS (clpS) (1 of 2)
	transcript_9466	GRMZM2G017532	0,854261177	0,297281833	0,004058567	0,040821373	PTHR12629//PTHR12629:SF8 - DIPHOSPHOINOSITOL POLYPHOSPHATE PHOSPHOHYDROLASE // SUBFAMILY NOT NAMED (1 of 5)
	transcript_102693	GRMZM2G120579	0,734333234	0,224663444	0,001080839	0,014851771	PTHR31096:SF16 - ACT DOMAIN-CONTAINING PROTEIN (1 of 1)
	transcript_14546	GRMZM2G010490	0,726499789	0,201336041	3,08E-04	0,005449425	PF11833 - Protein of unknown function (DUF3353) (DUF3353) (1 of 7)
	transcript_159949	GRMZM2G123200	-0,872327862	0,311260348	0,005069774	0,04833493	NA
	transcript_24021	GRMZM2G498656	-0,89919219	0,269004811	8,30E-04	0,01212537	PTHR23423//PTHR23423:SF15 - ORGANIC SOLUTE TRANSPORTER-RELATED // SUBFAMILY NOT NAMED (1 of 2)

	transcript_146637	GRMZM2G535623	-2,745257573	0,912603228	0,002628337	0,029349472	NA
S. italica	transcript_141712	Seita.6G069100	5,970518835	1,396096121	1,90E-05	5,34E-04	NA
	transcript_141710	Seita.6G069100	2,520370265	0,357673103	1,83E-12	2,52E-10	NA
	transcript_141709	Seita.6G069100	2,355013828	0,53440202	1,05E-05	3,21E-04	NA
	transcript_141706	Seita.6G069100	1,797992321	0,297669184	1,54E-09	1,23E-07	NA
	transcript_159982	Seita.1G020000	1,512914677	0,328794388	4,20E-06	1,49E-04	NA
	transcript_111333	Seita.1G020000; Seita.1G206100; Seita.1G287000; Seita.2G220000; Seita.5G013900; Seita.7G323800	1,216992852	0,273657459	8,70E-06	2,75E-04	NA
	transcript_132325	Seita.1G020000; Seita.1G206100; Seita.1G287000; Seita.2G220000; Seita.3G323000; Seita.5G013900; Seita.6G105800; Seita.7G323800	1,057200482	0,308441331	6,09E-04	0,009436029	NA
	transcript_188622	Seita.9G581100	-0,548742314	0,181295747	0,002471739	0,028033395	NA
	transcript_120233	Seita.2G160500	-6,404150648	1,702985907	1,70E-04	0,003350395	NA
